# Photophysics and Photochemistry of Canonical Nucleobases’ Thioanalogs: From Quantum Mechanical Studies to Time Resolved Experiments

**DOI:** 10.3390/molecules22060998

**Published:** 2017-06-18

**Authors:** Serra Arslancan, Lara Martínez-Fernández, Inés Corral

**Affiliations:** 1 Departamento de Química, Módulo 13, Universidad Autónoma de Madrid, Madrid 28049, Spain; arslancan.serra@uam.es; 2 Istituto Biostrutture e Bioimmagini-Consiglio Nazionale delle Ricerche, Via Mezzocannone 16, Napoli I-80134, Italy; 3 Institute for Advanced Research in Chemical Sciences (IADCHEM), Universidad Autónoma de Madrid, Madrid 28049, Spain

**Keywords:** nucleobase derivatives, thionation, interstate crossings, singlet oxygen, ab initio calculations, photoexcited dynamics, time resolved spectroscopy

## Abstract

Interest in understanding the photophysics and photochemistry of thiated nucleobases has been awakened because of their possible involvement in primordial RNA or their potential use as photosensitizers in medicinal chemistry. The interpretation of the photodynamics of these systems, conditioned by their intricate potential energy surfaces, requires the powerful interplay between experimental measurements and state of the art molecular simulations. In this review, we provide an overview on the photophysics of natural nucleobases’ thioanalogs, which covers the last 30 years and both experimental and computational contributions. For all the canonical nucleobase’s thioanalogs, we have compiled the main steady state absorption and emission features and their interpretation in terms of theoretical calculations. Then, we revise the main topographical features, including stationary points and interstate crossings, of their potential energy surfaces based on quantum mechanical calculations and we conclude, by combining the outcome of different spectroscopic techniques and molecular dynamics simulations, with the mechanism by which these nucleobase analogs populate their triplet excited states, which are at the origin of their photosensitizing properties.

## 1. Introduction

Thiobases are the result of substituting an exocyclic carbonyl oxygen atom by a sulfur atom in nucleobases. Despite their structural similarity to their canonical analogs, the photophysics of thiated nucleobases is dramatically different. In contrast to canonical nucleobases, which are able to repopulate the ground state (GS) in ultrafast time scales [1,2,3], the main relaxation pathways in thiated nucleobases drive the system to the most stable triplet excited state in a very efficient manner, that would eventually decay to the GS in longer time scales [4].

Aside from fundamental interest that would seek to understand how the nature of the substituents or their position along the purine and pyrimidine heterocycles [5,6] imprint the topography of the excited state potential energy surfaces (PESs) directing the photophysics of nucleobases, it has been recently recognized that specific thiated nucleobases could have been part of primordial RNA macromolecules [7,8]. Moreover, these systems are currently considered as very valuable prototypes for photosensitizers to be used in pharmacological applications, for their photosensitizing properties and structural similarity with canonical nucleobases, which facilitates their inclusion into parent DNA molecules.

In fact, 2-thiouracil (2t-Ura), 4-thiouridine (4t-Urd), 2-thiocytidine (2t-Cyd) and 2-selenouracil were found in transfer ribonucleic acids (tRNAS) of some bacteria such as *Escherichia coli* or *Bacillus subtiles*, yeasts and other higher organisms, and have been related to the improvement of the accuracy and efficiency of the translation process, fostered by the electronic properties and the size of S and Se elements [9,10]. Recent studies on non-enzymatic replication [7,8] have discovered that the exchange of Uracil (U) by 2t-Ura mends important problems along the replication process, such as the slow rate when copying RNA templates or the occurrence of G:T and A:C mispairs [7]. These interesting findings have been interpreted as an indication that thiated compounds might have played a role in primordial RNA replication processes, before the first RNA polymerase enzymes emerged.

As for their pharmacological properties, thiopurines, such as azathioprine, 6-mercaptopurine and 6-thioguanine (6t-Gua), have stood out for their clinical effectiveness as anticancer, anti-inflammatory or immunosuppressant prodrugs for decades [11,12]. Following enzymatic metabolization, a small fraction of these compounds is incorporated as 6t-Gua nucleotides into patients’ DNA undergoing these treatments [13,14,15]. This very small guanine by 6t-Gua substitution (below 0.02%) suffices, however, to increase the sensitivity of 6t-Gua containing DNA to UVA light. The absorbance of UVA radiation by 6t-Gua DNA would precipitate Type I and Type II mechanisms, leading to important photosensitized lesions such as canonical nucleobases’, 6t-Gua’s and protein’s oxidation products, DNA and DNA–protein crosslinks and DNA strand breaks [16].

Nucleobase thioderivatives, such as 4t-Urd, 4-thiothyimine (4t-Thy), and 6t-Gua, have also shown a great utility as photolabels to gain detailed knowledge on the structure of nucleic acids, as well as to recognize interaction points within nucleic acids or in nucleic acids protein aggregates, profiting their photocrosslinking properties upon UVA light absorption [17,18,19,20,21,22,23].

Finally, thiation has also been intensively employed in structural biology studies to understand the chemical, structural and functional alterations introduced by these substituents in the nucleic acids where they were incorporated [24,25,26,27,28].

In this scenario, it is not surprising that the literature on the photophysics and photochemistry of thionucleobases has experienced major growth, especially in the last decade, from both the experimental and computational sides, with the aim of unraveling the differences at the electronic structure level that distinguish these compounds from canonical nucleobases and deciphering the relaxation pathways underlying the very appealing photosensitizing properties of these systems for pharmacology and medicinal chemistry. Considering the abundant literature focused on particular aspects of the photophysics and photochemistry of thiated nucleobases, it is our aim here to compose a concise but complete picture, sewing together contributions from different fields, which summarizes not only the main optical properties of these systems, but also their main deactivation pathways.

As for canonical nucleobases, tautomer equilibria in thiobases have been intensively studied both from computational and experimental points of view. Thiouracils and their N1-methylated derivatives were found to be mainly present in their keto-thione forms, both in gas phase and in solution [29,30,31,32]. On the contrary, theoretical investigations on 2-thiocytosine (2t-Cyt) concluded that in vacuum its thiol form is more stable than the canonical tautomer, although the latter becomes strongly predominant in polar solvents [33,34]. Similar trends have been described also for the tautomeric equilibrium of 6t-Gua [35,36]. Since keto-thione is the predominant form for all thiobases in a biologically relevant environment such as water, most of the theoretical works have been performed on these tautomers and thus it will be on them where we will put the focus of our review.

We will also overlook recent works on the photophysical properties of Se- and Te-nucleobase derivatives [37,38], in line with those of thiated nucleobases, and the characterization of the electronic excited states of ionized thionucleobases, key to the interpretation of UV photoelectron spectra recently measured/simulated for some thiouracil derivatives [39,40].

The review is organized as follows. For all the thionucleobases considered, namely the thiopurine 6t-Gua, the thiopyrimidines 2-thiothymine (2t-Thy) and 2t-Ura, 4t-Thy and 4-thiouracil (4t-Ura), 2t-Cyt and 2,4-dithiothymine (2,4-dtThy), and 2,4-dithiouracil (2,4-dtUra), we will start by compiling the description of the experimental absorption and luminescence spectra and their interpretation with the help of Time Dependent Density Functional Theory (TD-DFT) and high level ab initio calculations. When discussed in the literature, we will briefly comment on the effect of the solvent and glycosylation on the main absorption and emission bands, by comparing with the absorption and emission spectra of the corresponding (deoxy)ribosides. Next, we will summarize the main features of the PES explored with the above-mentioned theoretical methods, sketching the most plausible relaxation mechanisms. Then, the photodynamics of these systems will be discussed based mainly on the results of transient absorption experiments, and in specific cases of time-resolved photoelectron and Resonance Raman spectroscopies. We will also comment on the fate of the triplet excited states, responsible for the photosensitizing properties of these systems. Detailed overviews on the fundamentals and performance of the main theoretical methods and spectroscopies discussed along this review can be found in Refs. [41,42,43,44,45,46,47,48,49].

Finally, the last section of the review concludes with some general remarks on the effect that the degree and position of thiosubstitution has on the absorption spectra, PES and photoexcited dynamics of thionucleobases and a very brief description of the main challenges in the field.

## 2. Canonical Nucleobases’ Thiated Analogs Photophysics

### 2.1. Thiopurines

#### 2.1.1. Steady State Absorption and Emission properties

**Figure 1:**
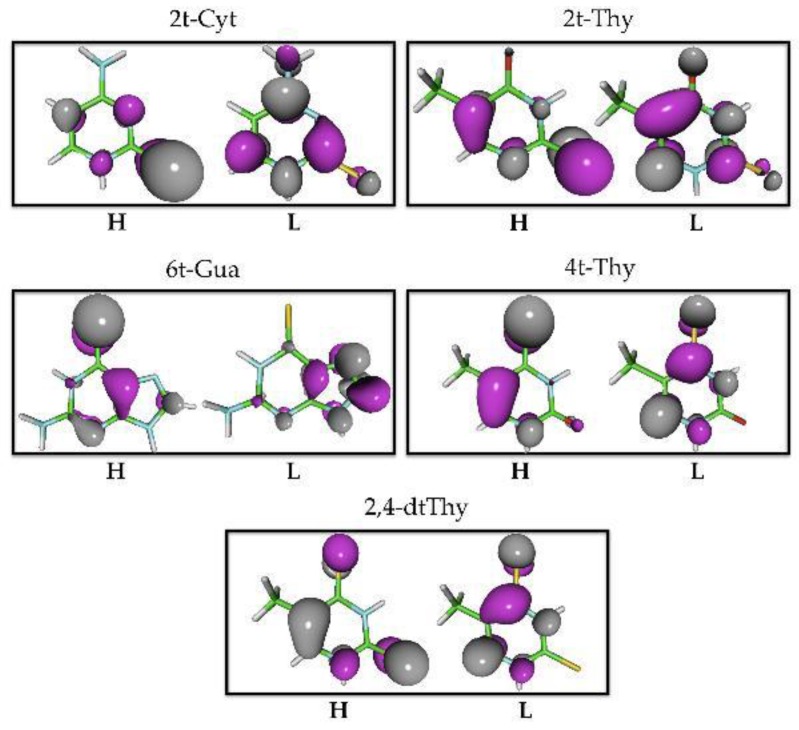
HOMO (H) and LUMO (L) molecular orbitals involved in the ππ* lowest spectroscopic state in 2t-Cyt, 2t-Thy, 6t-Gua, 4t-Thy and 2,4-dtThy.

Table 1 collects the experimental absorption and fluorescence emission maxima of 6t-Gua and its nucleosides 6-thio(2′-deoxy)guanosine (6t-Guo) in different solvents. Table 2 also summarizes in vacuum and in solution theoretical vertical absorptions and adiabatic emission energies calculated for 6t-Gua and 6t-Guo. According to these experiments, both the nucleobase and the nucleoside exhibit absorption maxima in the UVA region between 348–316 nm, (3.56–3.92 eV) for all the solvents examined, alkaline media providing the lowest wavelength values, as already noticed by Rubin et al. and Santhosh et al. [50,51]. Other peaks were found around 255, 220 and 205 nm in neutral and acidic conditions, whereas alkaline pHs induce a bathochromic shift in these absorptions which appear at 265, 245 and 210 nm [51]. DFT [38,52,53,54] and multiconfigurational approaches [55] predict the spectroscopic state in 6t-Gua, to absorb between 311–299 nm (3.99–4.15 eV) in gas phase (GP), whilst the computationally more restrictive semi-empirical CNDO (Complete Neglect of Differential Overlap) method [50,51] calculates this transition at significantly lower energies ca. 340 nm. All these methods, however, agree in assigning this transition a ππ* (Highest Occupied Molecular Orbital (HOMO) to Lowest Unocccupied Molecular Orbital (LUMO)) character and locate a very weakly absorbing lower-lying nπ* electronic state in the region 400–369 nm (3.10–3.36 eV). These two transitions are localized within the C-S bond (see Figure 1). TD-DFT predicts a shift of these two absorptions after including solvent-chromophore interactions, i.e., whilst the nπ* absorption is displaced to higher energies (ca. 0.4 eV) both in water and acetonitrile (ACN), the ππ* transition blue shifts by 0.1 eV in the same solvents. 

Discrepancies between the different theoretical approaches employed to predict the absorption spectrum of 6t-Gua are larger when predicting higher-lying excited states. Semi-empirical and TD-DFT methods locate a second ππ* excited state centered at ca. 295 nm (4.20 eV), absorbing with different intensities (0.0076–0.48), whilst the more reliable MS-CASPT2/CASSCF (Multi State Complete Active Space Perturbation Theory/Complete Active Space Self-Consistent Field) predicts a quite bright state at higher energies 253 nm (4.90 eV).

The comparison between the absorption features of 6t-Gua and its nucleosides (Table 1) reveals that glysosylation has very little effect in the position of the absorption maxima and only slightly increases the intensity of the UVA and UVC bands.

Fluorescence spectra of 6t-Gua recorded at different pH conditions and excitation wavelengths showed a band with a maximum peaking at 400–468 nm, with a shoulder around 500 nm [51,52]. Fluorescence decay was best fitted with two lifetimes in the range of ns, i.e., τ_1_ = 1.4 and τ_2_ = 24.1 ns [52]. 

Finally, phosphorescence signals centered in the range of 430–550 nm, depending on the pH conditions and on the experimental setup, were also registered [50,52]. Phosphorescence emission was found to decay with a lifetime of 45 ms [52].

#### 2.1.2. Static Description of the PES

**Figure 2:**
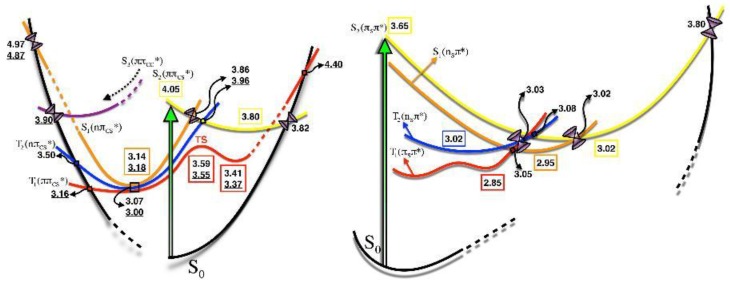
Potential energy profiles for 6t-Gua (**Left**) and 2t-Cyt (**Right**) inferred from quantum mechanical studies. Energies in eV relative to the ground state. For 6t-Gua, underlined energies were taken from Ref. [58]. The rest were extracted from Ref. [55]. For 2t-Cyt, energies were taken from Ref. [34]. The double well profile of T_1_ is based on Ref. [59].

A scheme of the potential energy profiles for 6t-Gua based on minimum energy path (MEP) calculations can be found in Figure 2.

**Scheme 1:**
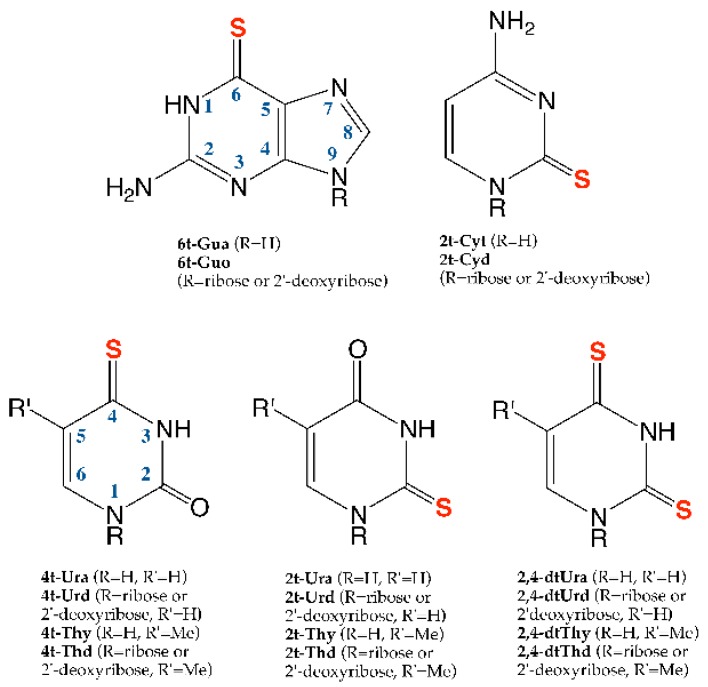
Structures of the thiosubstituted analogs of the nucleobases considered in this work. Atom numbering is given for the heterocyclic rings of the nucleobases.

The photophysics of 6t-Gua (6t-Guo) is dictated by the existence of two minima in the S_1_ potential, showing ππ_CS_* and nπ_CS_* characters, and lying 3.80 (3.5) and 3.14 (3.3) eV over the GS equilibrium structure, according to the MS-CASPT2//CASSCF [55] (TD-B3LYP [38]) calculations. These two minima are accessed via the S_2_(ππ_CS_*)/S_1_(nπ_CS_*) conical intersection (CoIn) in the vicinity of the Franck Condon (FC) region. Reaching these minima requires in 6t-Gua the lengthening of the C-S bond, besides the out-of-plane movement of the C-S bond in the nπ_CS_* minimum or the pyramidalization of the C2 (see Scheme 1 for atom labeling) coupled to the rotation of the amino group in the ππ_CS_* minimum [55]. The S_1_ and S_2_ (corresponding to S_1_(ππ_CS_*) in Ref. [55]) minima of the deoxynucleoside were found to experience parallel geometrical C-S distortions as those reported for the thionucleobase. No significant out-of-plane deviation of the NH_2_ group was, however, registered for 6t-Guo [38]. Similar geometries were obtained for equivalent minima of triplet multiplicity located 3.41 (^3^ππ_CS_*) eV and 3.07 (^3^nπ_CS_*) eV relative to the GS minimum in 6t-Gua [55]. A lower energy (2.5 eV), however, was registered for the most stable T_1_ minimum of 6t-Guo [38]. Interestingly, a third triplet minimum with ππ_CS_* character, identical geometry and thus energy to the nπ_CS_* singlet and triplet minima was also optimized for the thionucleobase [55].

Although a one to one comparison between the calculated adiabatic emission energies and the fluorescence and phosphorescence (Table 1 and Table 2) signals is not possible, since the vibrational structure of the ground and S_1_ excited state was not considered by the available calculations, the energies calculated for the most stable singlet and triplet minima are consistent with the luminescence signals experimentally obtained. 

The same multiconfigurational study [55] locates for 6t-Gua two S_1_/S_0_ internal conversion (IC) funnels for the return of population to the S_0_ from the ππ_CS_* and nπ_CS_* minima. Reaching these funnels requires the perpendicular arrangement of the C-S bond to the purine ring, and the pyramidalization of several C and N centers in the case of the S_1_(nπ_CS_*)/S_0_, importantly affecting the energy of this interstate crossing point which lies almost 2 eV above the nπ* minimum. The S_1_(ππ_CS_*)/S_0_ crossing, with almost the same energy as the ππ_CS_* minimum, is in contrast easily accessible for the S_1_ potential. A third S_1_(ππ_CC_*)/S_0_ IC funnel, lying at intermediate energies (3.9 eV), was identified with the help of molecular dynamics (MD) simulations on photoexcited 6t-Gua [58]. This interstate crossing is geometrically characterized by the puckering of the C7 and the folding of the NH_2_ group, and is accessible after surmounting a 0.72 eV energy barrier from the ^1^nπ_CS_* minimum.

The two S_1_ minima, lying both structurally and energetically very close to the minimum energy crossing points (MECP) S_1_(ππ_CS_*)/T_2_(nπ_CS_*) and S_1_(nπ_CS_*)/T(ππ*), were identified as efficient funnels for transferring the population to the triplet manifold, due to the proximity of strongly coupled triplet states (calculated spin-orbit couplings (SOC) of 200 cm^−1^). Once in the triplet manifold, the system needs to climb energy barriers comprised between 0.1 and 1 eV to access intersystem crossing (ISC) funnels for further decaying to the S_0_. The SOCs calculated at these regions are small (3–12 cm^−1^), except for the T_1_(nπ_CS_*)/S_0_ crossing, which was predicted to amount to 270 cm^−1^. It is important to note here that all the stationary and interstate crossing points were connected with MEP calculations.

#### 2.1.3. Photodynamics

**Figure 3:**
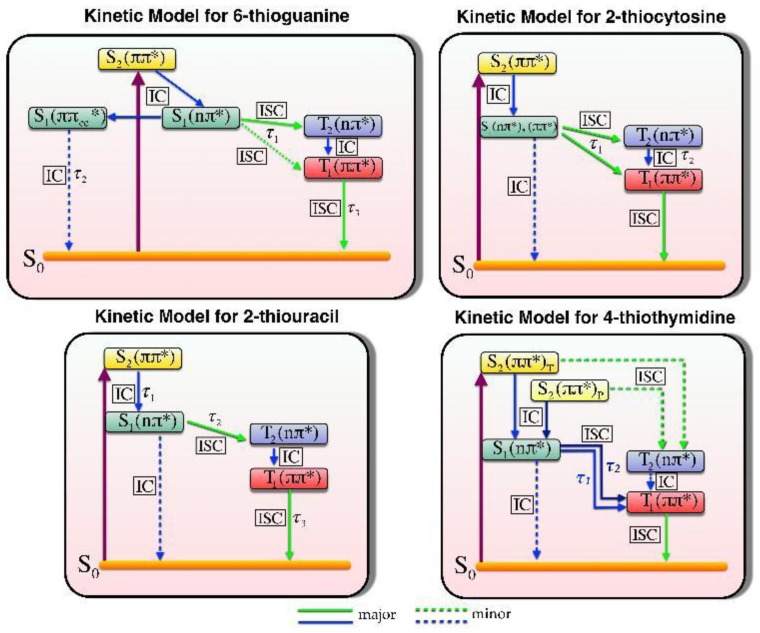
Proposed kinetic models for nucleobase thioderivatives inferred from the literature.

The excited-state dynamics of the nucleoside 6t-Guo and the nucleobase 6t-Gua were respectively studied by means of femtosecond broadband transient absorption spectroscopy (TAS) [52,54] and singlet/triplet surface hopping dynamics using a parameterized semi-empirical Hamiltonian with CASPT2 calculations [58]. The excited state dynamics of the nucleoside (nucleobase) is governed by a single excited state species showing two transient absorption signals at 380 (375) and 620 (520) nm, which evolve in parallel at early time delays and remain stationary after 2 ps. This transient is assigned to a triplet excited state, based on the comparison of the decay lifetimes of the absorption signals in N_2_ and air-saturated conditions, which are 1.5 times increased in the absence of oxygen [54]. The triplet species in the nucleoside is populated in ultrafast timescales τ_1_ = 0.31–0.36 ps, both in aqueous solution and in ACN (See Figure 3, and Table 3). For the nucleobase, however, this process was measured to occur in longer time scales τ_1_ = 0.56 ps [52].

A second lifetime for the nucleoside (nucleobase) τ_2_ = 32–80 (26) ps was also fitted from the dynamics and assigned to the minor repopulation of the GS from the S_1_(nπ_CS_*) excited state combined with solvation dynamics of the T_1_ (See Figure 3, Table 3) [54]. Finally, the longer lifetime in 6t-Guo (6t-Gua) τ_3_ = 720 (830–1420) ns is ascribed to the ISC process from the triplet manifold to the GS [52,54].

From the above data, it can be concluded that the effect of N9-glycosylation is not homogenous along the dynamics of the chromophore. While N9-glycosylation accelarates the ISC processes for the population of the triplet manifold and the GS, it noticeably slows down the IC process S_1_→S_0_.

The complex topography of the excited state PES of thiobases invalidates the interpretation of the photodynamics of these systems based on the FC picture and, moreover, urges the use of MD simulations due to the existence of several competing relaxation pathways [54,58]. The results from the singlet/triplet MD simulations [58] are in line with the experimental results [52,54] and consistent with the topography of the PES predicted by static quantum chemical methods [55]. In the dynamics simulations, the S_2_ internally converts in ultrafast timescales to the S_1_. The depopulation of the S_1_ in favor of the T_1_ and T_2_ states is also ultrafast. Only very few direct hops were observed from the S_2_ to the triplet manifold. The very small population (0.15%) that returns back to the GS, during the 10 ps that the simulations last, proceeds via the S_1_(ππ_CC_*)/S_0_ CoIn which is accessed from the S_1_ nπ_CS_* minimum after overcoming a small energy barrier through which the S_1_ electronic state evolves and adopts a ππ_CC_* character (Recall Figure 2). Interestingly, the dynamics is able to capture critical features of the topography of the PES, such as the degeneracy between the S_1_, T_1_ and T_2_ electronic states around the region of the most stable S_1_ minimum, where the system gets trapped during some time. The energetic proximity of these three electronic states translates into multiple recrossings among them along the trajectories examined. At the final time of the simulations, the population is fundamentally confined in the T_1_ electronic state, with some excited 6t-Gua molecules in the T_2_ and residual population distributed between the S_1_ and S_0_ [58]. The fitted lifetimes extracted from the MD simulations and which amount to τ_1_ = 122 fs and τ_2_ ≈ 200 ps are of the same order of magnitude as the experimental results obtained for the 6t-Gua and 6t-Guo. 

Finally, a very high triplet yield of 0.9 [58] was inferred from the MD simulations, which is slightly higher than the experimental value of 0.6–0.8 [52,54].

#### 2.1.4. The Fate of the Triplet Excited States of 6t-Gua and 6t-Guo

The very extended use of thiopurine prodrugs, which are enzymatically converted to 6t-Gua nucleotides before being incorporated into DNA, as immunosuppressants, anti-inflammatory and anticancer drugs greatly contributed to intensifying the research on the fate of the triplet excited state of this nucleobase and its nucleoside to better understand their mechanism of action. The chemical evolution of these triplet intermediates importantly depends on the environment where the nucleobase derivative is immersed and where it is expected to undergo Type I and Type II photosensitization reactions, leading either to cellular lesions, if 6t-Gua happens to be incorporated in a DNA macromolecule or to potential toxic products if photosensitization takes place in solution.

In fact, Type I mechanisms rely on the reaction of the photosensitizer with a substrate to generate substrate’s and photosensitizer’s radical species, via hydrogen abstraction or electron transfer processes, that would eventually evolve to oxygenated products or would produce the superoxide radical O_2_^−^ following an electron transfer process in the presence of molecular oxygen. 

Type II photosensitization, in turn, involves an energy transfer process, where triplet excited 6t-Gua transfers its excess of energy to molecular oxygen from the environment, which is excited to singlet oxygen, with the concomitant return of the thiobase back to the GS.

Zhang et al. determined in 2011 the quantum yield of ^1^O_2_ production (Φ_Δ_) for 6t-Gua and 6t-Guo, by following the oxidation of the water soluble phosphine, [2-(dicyclohexylphosphino)ethyl]trimethylammonium chloride by ^31^P NMR [56]. These authors obtained, depending on the pH conditions, Φ_Δ_ values which oscillated within 0.56–0.58 for 6t-Gua and 0.49–0.55 for 6t-Guo (see Table 3). Only recently, Pollum et al. estimated the ^1^O_2_ quantum yield for 6t-Guo using ^1^O_2_ phosphorescence and obtained significantly lower values compared to previous studies, Φ_Δ_ = 0.24–0.29 [73]. These unexpectedly low Φ_Δ_, considering the high triplet quantum yields measured for these systems, were ascribed to the low values determined for the fraction of quenching events (S_Δ_), which for 6t-Guo only amounts to 0.37, suggesting the concurrence of other competing events [73].

**Scheme 2:**
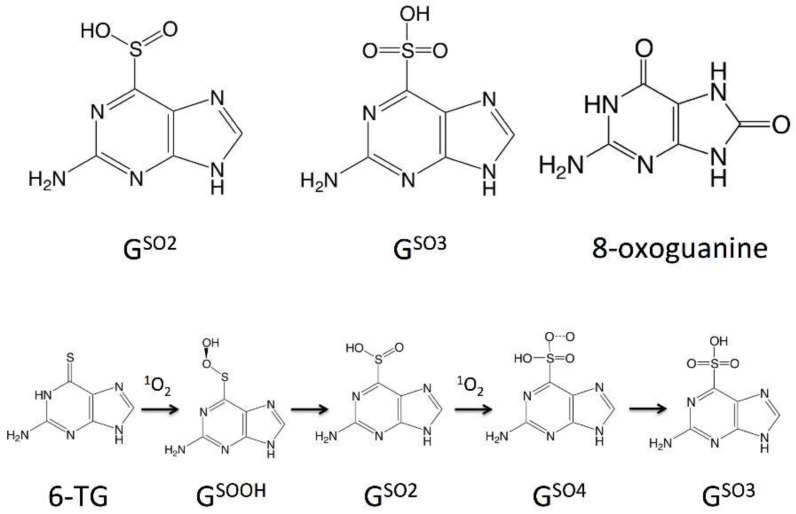
Oxidation products of 6-thioguanine and guanine (**Top**); and their generation mechanism according to Ref. [77] (**Bottom**).

The ^1^O_2_ produced from Type II photosensitization reactions has been postulated to be involved in the oxidation reactions of 6t-Gua, canonical nucleobases and proteins that can be considered as major routes for DNA and/or cellular damage [16]. The main oxidation products of 6t-Gua are guanine-6-sulfinate (G^SO2^) and guanine-6-sulfonate (G^SO3^) [15,56,74]. Interestingly, guanine is the canonical nucleobase more prone to oxidation, producing 8-oxoguanine (8-oxo-7,8-dihydroguanine, See Scheme 2) [75]. The production of ^1^O_2_ has also been found to induce histidine-lysine crosslinking processes in subunits of the PCNA replication and repair complex [76].

In 2013, Zou and coworkers investigated, by combining DFT calculations and experimental measurements of reaction stoichiometry and rate constants, the mechanisms by which the mutagenic G^SO2^ and G^SO3^ products are generated [77]. Specifically, these authors invalidate the mechanism, which postulates guanine sulfenate (G^SO^) as an intermediate in the formation of G^SO2^ and G^SO3^ products [15,74,78], and propose instead the more energetically favored formation of G^SO2^ through a peroxy intermediate (G^SOOH^), accessed after H transfer from the NH group of the pyrimidine ring towards the terminal O atom of the 6t-Gua-^1^O_2_ complex. This peroxy intermediate would then evolve in a second stage to G^SO2^ product, after the migration of the OH group from the O to the S center (see Scheme 2). Further oxidation of G^SO2^ by ^1^O_2_ would lead to G^SO4^ and to the final oxidation product G^SO3^ with the assistance of a water molecule. The very low barriers theoretically predicted for the oxidation 6t-Gua→G^SO3^ are supported by the fast rate constant measured, which amounts to 4.9 × 10^9^ M^−1^ s^−1^. These authors additionally mapped at molecular level a secondary pathway competing with the formation of G^SO2^ which explains the residual formation of Guanine + sulfur monoxide, SO, in low yields, due to the larger energetic barrier involved.

The complexity of other UVA-photoinduced 6t-Gua photoproducts, such as intra- and interstrand [79] DNA crosslinking, DNA breakage [80] or DNA–protein crosslinking [17,81,82], has prevented the in detail study of the mechanism behind the formation of these photolesions, remaining in some cases the role played by Type I and Type II photosensitization reactions still unknown. In fact, some recent works on 6t-Gua and 6t-Guo have demonstrated, based on theoretical grounds, that electron transfer (Type I mechanisms) reactions between the triplet excited states of 6t-Gua or 6t-Guo and ground state molecular oxygen are energetically favorable, and might therefore offer a plausible explanation for the reduced singlet oxygen quantum yield of this chromophore [73,83]. In fact, Pollum et al. estimate the value of ΔG for the electron transfer reaction in 6t-Guo to amount to −0.72 eV, using the Rehm-Weller equation and the vertical ionization energy of 6t-Guo predicted by DFT calculations in solution [73]. A moderately lower value (−0.26 eV) was calculated in Ref. [83] considering the complex formed between the triplet excited state of 6t-Gua and ^3^O_2_ and using the CASPT2//CASSCF protocol, including solvent solute interactions via the Polarizable Continuum Model (PCM) model.

### 2.2. Thiopyrimidines

#### 2.2.1. 2-Thiopyrimidines

##### 2t-Cyt/Cyd

• Steady State Absorption and Emission properties

The absorption spectra of 2t-Cyt and 2t-Cyd in water were recorded in the early 1980s, showing both a maximum at ca. 270 nm (~4.59 eV, see Table 4) [84]. More recently, the absorption spectrum of 2t-Cyt has been revisited confirming the presence of a maximum at 270 nm (Band I) and ascertaining the existence of a second peak at ~240 nm (Band II, 5.17 eV) and a shoulder ~220 nm (5.64 eV) [34,85]. The two bands are affected in different ways by the change of the polarity and the nature of the solvent from polar-protic (water) to polar-aprotic and less polar-aprotic solvents (DMSO) [85]. Whereas Band I is red-shifted by ~15 nm, but maintains its intensity, when moving from water to DMSO, the position of Band II is less affected but significantly decreases its intensity, vanishing in DMSO [85].

GP MS-CASPT2 calculations (Table 5) for 2t-Cyt predict the first bright state S_2_ (π_S_π*) at ~330 nm (3.76 eV), which has been connected with the low-energy tail of Band I at 270 nm. The absorption maximum of Band I has been ascribed to the second bright state S_4_ (π_S_π*) (~4.30–4.40 eV) [34,85]. The oscillator strengths computed for both states confirm this assignment, since the S_2_ (π_S_π*) is at least five times less intense than S_4_ (π_S_π*) [34,85]. The S_1_ (n_S_π*) corresponds instead to a dark excited state [34,85]. Higher in energy bright states, S_6_ and S_8_, have been recently proposed by Mai et al. to contribute to Band II [85]. These authors have paid especial attention to the performance of MS-CASPT2 in the prediction of solvated absorption spectra and their comparison with the experiment. The computed dipole moments of the electronic excited states together with results from explicit solvent-solute calculations were used to explain the shift of the transitions induced by the solvents: S_4_ (π_S_π*) with a smaller dipole moment than the GS was found to blue-shift in polar solvents, whereas the opposite was found for S_6_ and S_8_, in agreement then with the experimental observations [85]. This blue-shift of the first absorption band (arising from a ^1^ππ* excited state) in water was previously reported for other purine/pyrimidine bases [5,87,88]. However, the accurate reproduction of the experimental changes in the band intensities was found to be more challenging [85].

• Static Description of the PES

After excitation at 310–320 nm wavelengths, which are the ones used in femtosecond transient absorption experiments, the S_2_ (π_S_π*) is mainly populated. This excited state is expected to rapidly decay in favor of two lower-lying S_1_ minima with ^1^π_S_π* and ^1^n_S_π* characters. Indeed, GP MS-CASPT2 MEP calculations reveal that the IC funnel allowing the S_2_→S_1_ decay is reached barrierlessly from the FC region, see Figure 2 [34]. Both singlet minima maintain the pyrimidine ring planarity but show some differences for particular bond lengths, the most significant concerns the C5–C6 bond that is larger for the ^1^π_S_π* minimum. Besides being geometrically similar, MS-CASPT2 also predicts excited state features common to both minima. First, they are stable against GS repopulation, since the IC funnels with the S_0_ were located significantly high in energy (~0.8 eV) over the corresponding minima [34]. By contrast, the presence of close lying singlet/triplet MECP (^1^π_S_π*/^3^n_S_π* or ^1^n_S_π*/^3^π_S_π*), where large SOC were calculated (160 cm^−1^), identifies these singlet minima as ideal regions of the PES for singlet→triplet population transfer [34]. Thereby, 2t-Cyt does not present any long-lived singlet minima and, although not having yet been reported, the experimental fluorescence quantum yields are expected to be negligible.

Once in the triplet manifold, a CoIn between the two triplet states, ^3^π_S_π*/^3^n_S_π*, was located close in energy to both singlet-triplet MECPs (^1^π_S_π*/^3^n_S_π* and ^1^n_S_π*/^3^π_S_π*). Thus, according to MEP calculations both triplet minima (^3^n_S_π* or ^3^π_S_π*) could be a priori populated (see Figure 2). Both triplet minima structurally resemble their singlet counterparts. Non-radiative GS repopulation from these triplet minima seems also unlikely due to the presence of large energy barriers to access the ISC funnels [34]. Unfortunately, no experimental phosphorescence measurements for 2t-Cyt are so far available.

Two different ^3^π_S_π* minima have been recently characterized by Bai et al. at CASPT2 level for 2t-Cyt (Figure 2). Similarly to 6t-Gua, the two minima differ in their geometries and in their decay mechanisms. Although the relative stability of the two 2t-Cyt triplet minima has not been discussed, the first shows a clear out of plane deviation of the S atom and is expected to easily decay to the GS, whereas the other presents a ring-distorted structure and is expected to live longer [59].

• Photodynamics

2t-Cyt has also been investigated by means of femtosecond TAS using a 308 nm excitation wavelength [34]. For 120 fs time delay onwards, the TAS shows two different absorption maxima, at 350–400 nm and at ~600 nm. For a time delay of 320 fs, the former reaches its maximum whereas the latter blue-shifts (to ~525 nm) and continues to grow also in the ps time regime, being its decay not observed within 20 ps. Two different time constants in the fs regime were found to correctly fit these TAS, τ_1_ = 210 fs and τ_2_ = 480 fs and a close-to-unity triplet yield was reported (see Table 3, Figure 3) [34].

GP Multi Reference Configuration Interaction Singles (MRCIS) dynamics simulations showed a fast decay of the 2t-Cyt S_2_ population, in 160 fs, in agreement with the absence of stable minima along the S_2_ PES. An average ISC time constant of 250 fs was calculated, which is in good agreement with the experimental value of τ_1_ = 210 fs (See Figure 3) [34]. Although it is not possible to discern between the two different ISC routes (^1^π_S_π*/^3^n_S_π* or ^1^n_S_π*/^3^π_S_π*), MRCIS dynamics predicts that depopulation from the S_1_ to the T_2_ is preferred. Thus, the first time constant would account for the first deactivation step involving S_2_→S_1_→T_2_ relaxation [34]. Once in the triplet manifold, subsequent T_2_→T_1_ IC takes place, holding T_1_ most of the population from 400 fs onwards. Thus, the second experimental time constant τ_2_ = 480 fs was ascribed to IC in the triplet manifold [34].

Despite their structural similarities, the ^1^π_S_π* and ^1^n_S_π* minima present very different absorption patterns. According to MS-CASPT2 calculations, they respectively absorb at 360 and 600 nm, with different intensities, the ^1^π_S_π* minimum presenting much larger absorption [34]. Thereby, the comparison of experimental TAS at representative time delays with computed vertical absorptions at relevant points of the excited PES has been used to provide further mechanistic information on 2t-Cyt photoinitiated dynamics. As described above, for a 320 fs time delay, the experimental TAS reaches a maximum at ~350 nm and presents some absorption also at ~600 nm, which Mai et al. attribute to a ~10% and ~25% population distribution between the ^1^π_S_π* and ^1^n_S_π* minima, respectively [34]. This residual population at the singlet minima at 320 fs is in agreement with the ISC lifetime (200 fs); in other words for these timings most of the molecules have reached the triplet manifold [34]. For longer time delays, ~4 ps, the recorded experimental TAS is blue-shifted in agreement with the MS-CASPT2 vertical absorption computed for the ^3^π_S_π* minimum centered in the 500–550 nm region [34].

2t-Cyt presents stable long-lived triplet minima, which are both efficiently and rapidly populated from short-lived singlet excited states.

##### 2t-Thy/Thd and 2t-Ura/Urd

• Steady State Absorption and Emission properties

In aqueous solution, the experimental absorption spectrum of the nucleobase 2t-Ura covers the range 360–240 nm (3.44–5.17 eV) with a maximum of absorption around 265 [61]–274 [84] nm (4.68–4.52 eV, Table 4). Interestingly, the absorption band slightly modifies its shape when recorded in ethanol, methanol and ACN. In ethanol or methanol, it presents an additional shoulder at 291 nm (4.26 eV) that increases its intensity when switching the solvent to ACN [84,86]. According to experiments, glycosilation was found to have no effect on the transition coinciding with the absorption maximum S_2_ (ππ*), however it shifts by 0.05–0.1 eV to higher energies the S_1_ state and redshifts by 0.2 eV the S_3_ [84].

Methylation at position 5 has also very little effect on the absorption properties. In fact, 2t-Thy shows maximal absorption around 275 nm in phosphate buffer solution (PBS), whilst two absorption maxima at 275 and 290 nm, with different relative intensities depending on the solvent used, were registered when recording the spectrum in ACN or ethanol [62,63,86]. As for 2t-Ura, Glycosilation only shifts by 2–10 nm the absorption maximum to lower energies, whilst significantly increasing the value of the molar absorptivity coefficient [63,64]. Different quantum chemical methods have been employed to predict the absorption spectrum of these systems, which cover from monoconfigurational DFT [31,37,62,86,90,92] to multiconfigurational [89,90,91] or multireference [89,93] approaches, such as CASPT2/CASSCF and MRCI (See Table 5). For 2t-Ura, these methods predict in GP the lowest lying brightest excited state S_2_ (π_S_π_456_*), (the subscripts refer to the pyrimidine centers where the π* orbital is localized), which promotes an electron from the HOMO mainly localized on the S atom to the LUMO extended over the C4-C5-C6 atoms, in the range between 304–215 nm (4.08–5.77 eV) (recall Scheme 1 and Figure 1). This transition is ascribed to the shoulder at 4.26 eV of the experimental spectrum, which becomes apparent in solution [84]. From the wide variety of methods employed in the simulation of the absorption spectrum of 2t-Ura, MRCIS is the one that provides the largest deviation compared to the others. The poor performance of this method has been ascribed to the importance of higher excitations or the use of a larger reference space in the correlation treatment for the correct description of the electronic transitions in this system [89].

Solvent solute interactions included using the PCM model shift this transition by 0.25 eV to higher energies, both for water [31] and ACN [31,92]. All these electronic structure methods also calculate two very weak nπ* transitions flanking the spectroscopic state. The first transition, localized at the C-S bond, was found to be centered ca. 347–322 nm (3.57–3.85 eV). The second, however localized on the C-O bond, was found to peak ca. 300–260 nm (4.13–4.77 eV). These two transitions are also displaced to higher energies when chromophore-water or chromophore-ACN interactions are considered [31,92]. Finally, a second quite intense ππ* transition (π_S_π_2_*), transferring electron density from the C-S bond to the C-O bond and importantly contributing to the absorption band, was calculated in the region 278–243 nm (4.46–5.10 eV). The effect of introducing a methyl group in position 5 (2t-Thy) leads to a blue shift of the calculated electronic transitions, by 0.1–0.4 eV in the case of the first n_S_π_2_* transition and ca. 0.1 eV or less in the case of the lowest lying π_S_π_456_* [62,86,93]. In a water continuum, the S_1_ and S_2_ transitions in 2t-Thy were predicted at 3.81 and 4.30 eV by the TD-M06 formalism [37]. MS-CASPT2, however, predicts the second π_S_π_2_* to absorb at the same position for 2t-Ura and 2t-Thy, although shifts to higher energies the second n_O_π_6_* in 2t-Thy, inverting the ordering of these two consecutive states [93].

Kuramochi et al. [62], Taras-Góslińska et al. [63] and Vendrell-Criado et al. [86] studied emission in 2t-Thy nucleobase and its riboside and deoxyriboside. Very small or no fluorescence quantum yields (<10^−4^) were detected for 2t-Thy [62,86]. Phosphorescence of the nucleobase and the nucleoside in 2-methyltetrahydrofuran (THF) and dichloromethane–methanol (DCM:MeOH) glassy matrices at 77 K and ethanol (EtOH) or deoxygenated ACN solution at room temperature was observed, with maxima at 454 [62], 448 [63], 451 [86], and 480 [63] nm, respectively. A similar value, at 427 nm [86], was obtained for 2t-Ura in ethanol. Phosphoresce quantum yields were also estimated to be very low (5 × 10^−4^) [63].

• Static description of the PES

**Figure 4:**
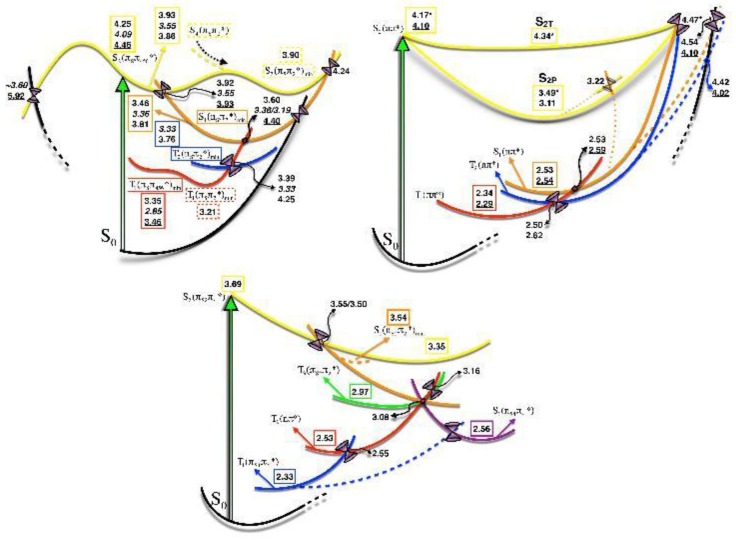
Potential Energy Profiles for 2t-Ura (**Top left**) and 4t-Thy (**Top right**) and 2,4-dtThy (**Bottom**) inferred from quantum mechanical studies. Energies in eV relative to the ground state. For 2t-Ura, underlined energies were extracted from Ref. [90], italic from Ref. [91] and the rest from Ref. [89]. For 4t-Thy, underlined energies were taken from Ref. [95] and the rest were extracted from Ref. [83]. The energies signaled with an asterisk denote geometries optimized including seven water molecules. For 2,4-dtThy, energies were taken from Ref. [96].

2t-Ura and 2t-Thy present a complex excited state PES whose main features are summarized in Figure 4. As a first approach to the problem, Gobbo et al. [91] and Cui et al. [90] locate, by means of CASSCF methods, two minima in the S_2_ and S_1_ potential preserving the ππ* and nπ* characters of the FC region (See Figure 4). At this point, it is important to highlight that all the works on 2t-Ura seem to provide a different FC state ordering [89,90,91]. According to Refs. [90] and [91], both minima are characterized by a significant elongation of the C2-S bond. However, whilst Ref. [91] predicts moderate out-of-plane distorted structures for both minima, caused by the pyramidalization of the C6 center, the Cs symmetry constraints imposed in the work of Cui prevent accessing the real minima [90]. A second additional minimum in the S_2_ potential, with π_S_π_2_* character, but corresponding to the FC S_4_ electronic state, is latter identified by the group of González [89], using CASPT2 gradients. This minimum maintains the stretched C-S bond distance but shows a strong pyramidalization at the C2 position. Interestingly, González [89] (2t-Ura) and Barbatti (2t-Thy) [93] predict a very similar strongly C2 pyramidalized structure for the S_1_ n_S_π_2_* minimum, in contrast with the quasi-planar structure calculated by Gobbo and Cui [90,91]. In the triplet manifold, altogether three triplet minima have been identified. Gobbo et al. and Cui et al. locate two minima with n_S_π_2_* and π_S_π_456_* characters in the T_2_ and T_1_ potentials (See Figure 4). Similar to what happened for the singlet manifold, CASPT2 predicts for the triplet n_S_π_2_* minimum a very important out-of-plane deviation of the C-S bond relative to the pyrimidine ring. Two minima with π_S_π_2_* and π_S_π_456_* characters along the T_1_ potential, the first pyramidalized at the C2 position and the second showing a boat like conformation were also found [89,93]. The adiabatic relative energies of the most stable triplet states of 2t-Ura, which were calculated in the range 2.85–3.46 eV, compare very reasonably with the experimental 0-0 emission phosphorescence band from Ref. [94] at 3.17 eV. This is also the case of 2t-Thy where the computed energies for the two ππ* T_1_ minima amount to 3.20 and 3.23 eV [59], which compare reasonably well with the maxima of the experimental phosphorescence spectra at 2.73–2.76 eV [62,63].

The former stationary points were connected by means of MEP calculations. Two S_2_/S_1_ crossings were optimized for each of the two ππ* S_2_ states to locate the IC funnels for the transfer of population from the spectroscopic state S_2_ to the lower lying S_1_. The S_2_(π_S_π_456_*)/S_1_ CoIn was found to lie close to the position of the S_2_(π_S_π_456_*) minimum, therefore being energetically accessible from this stationary point [89,90,91]. The S_2_(π_S_π_2_*)/S_1_ CoIn instead lies 0.35 eV above the S_2_(π_S_π_2_*) minimum becoming less accessible compared to the former. These theoretical works also considered the IC funnels for the return of population to the GS. Authors in refs. [90,91] compute the S_2_(π_S_π_456_*)/S_0_ CoIn respectively ca. 0.05 and 2 eV over the S_2_ minimum. Moreover, Gobbo et al. [91] predict an energetic barrier to access the S_2_/S_0_ CoIn, adding further difficulty to accessing this funnel. Mai et al. fail to optimize the S_1_(n_S_π_2_*)/S_0_ degeneracy point, and assume it should not play an important role in the photodynamics of 2t-Ura on the basis of the very large experimental ISC yields. Transfer of population to the triplet manifold has been postulated to take place both from the S_2_ and from the S_1_ potentials. Gobbo et al. [91] and Cui et al. [90] locate close lying triplet excited states, showing nπ* characters, at the position of the S_2_ (π_S_π_456_*) minimum, where they calculate moderate to large SOCs (20–190 cm^−1^), in line with El Sayed propensity rules [97]. More probable is, however, the leak of population to triplet potentials at the position (in the vicinity) of the S_1_ minimum where the calculated SOCs amount to 100–275 cm^−1^ [89,90,91,93]. Finally, the groups of Fang [90] and Barbatti [59] also explored the paths for the return of the population from the T_1_ potential to the S_0_. Cui and Fang (2t-Ura) compute a very high T_1_/S_0_ degeneracy points ca. 1.9 eV above the triplet minimum [90]. Bai and Barbatti (2t-Thy) instead calculate this ISC point only 0.3 eV above the C2-pyramidalized T_1_ minimum.

The complex topography of the excited state PES with multiple possible relaxation pathways called for a dynamics study on this system in order to learn more about the real deactivation mechanism.

• Photodynamics

The dynamics of 2t-Ura and 2t-Thy have been studied using nanosecond to femtosecond transient absorption experiments [60,61,62,63,64], Resonance Raman spectroscopy [92], time-resolved photoelectron spectroscopy [39] and ab initio singlet/triplet non-adiabatic MD simulations [98]. As for 6t-Gua, the dynamics of the 2-thiosubstituted derivatives of Thymine and Uracil is also dominated by a single excited state species absorbing in the visible and UVA regions, which rises in ultrafast time scales, and which shows absorption maxima around 350, 420 and 700 nm. [60,62,63,86] These values, that can slightly vary depending on the specific nucleobase and the solvent considered, are in qualitative agreement with the vertical transient spectra calculated by Bai and Barbatti at DFT/MRCI level of theory, confirming the population of the C2-pyramidalized and boat ππ* triplet minima along the dynamics [59]. The TAS of the ribosides were found to be very similar to that of the nucleobase [63]. This transient absorption signal is preceded by another UV signal (345 nm) populated within few hundreds of femtoseconds and which has been attributed to the absorption of the S_1_(nπ*) transient [60]. The photodynamics of these species can be then summarized in terms of the following lifetimes τ_1_(2t-Thy, 2t-Ura) ≤ 200 fs, τ_2_(2t-Thy) = 0.62 (aqueous solution), 0.32 (ACN) ps and τ_2_(2t-Ura) = 0.35 (aqueous solution), 0.34 (ACN) ps [60,64], and τ_3_(2t-Thy) = 2.7 μs, which were respectively assigned to the population of the S_1_(nπ*), the ISC lifetime and the triplet lifetime of these two nucleobases (See Table 3 and Figure 3) [62,63]. Glycosilation of 2t-Thy was found to reduce by a factor of 1.5 the ISC lifetime [64] and by a factor of 14–22 the lifetimes of the T_1_ [63], which the authors suggest can be ascribed to the higher density of vibrational states in the nucleosides. 

Notably, different time constants for 2t-Ura as compared to previous works in solution were determined by GP time resolved photoionization measurements [39]. According to TR-PES experiments in GP, the S_1_ would be populated faster (τ_1_ < 100 fs) than in solution. The time constant extracted from the TR-PES spectra for the S_1_ ISC process is of the same order as the one calculated for 2t-Thy (τ_2_ = 775 fs). Quite surprisingly, ISC process for the repopulation of the GS seems to take place remarkably faster (τ_3_ = 203 ps) compared to other works considering similar systems in solution [60,62,63,86].

MD, using CASPT2 as the electronic structure method for the calculation of the energies, the gradients and the couplings, were undertaken to shed some mechanistic light on the ISC process [98]. 

In agreement with the experimental results, the dynamics simulations predict S_2_→S_1_ IC process and ISC to the triplet manifold in a few hundreds of femtosecond. From all the possible pathways to transfer population to the triplet manifold, the dynamics revealed the S_1_→T_2_ route as the main one (40%), followed by the S_1_→T_1_ (35%) and S_2_→T_2_ (25%). The lifetimes fitted from the dynamics simulations, τ_1_ = 59 fs, τ_2_ = 400 fs, and the triplet quantum yield of Φ_T_ = 0.90, are also in line with the experimental findings.

The MD simulations also revealed important features of the relaxation mechanism. For instance, most of the trajectories visit regions of the S_2_ potential corresponding to the planar π_S_π_456_* minimum, with only few of them accessing C2-pyramidalized regions of the PES, characteristic of the π_S_π_2_* minimum. In contrast, trajectories were found to distribute between the T_1_ boat conformation and the C2-pyramidalized T_1_ minima, the second carrying a larger population. The simulations are not able to properly describe the repopulation of the GS from the triplet manifold, due to the very small number of trajectories reaching the GS at the end of the simulation, which is also consistent with the experimental results.

Finally, intersystem crossing and singlet oxygen quantum yields have been also estimated for this family of compounds. The very high triplet quantum yields for 2t-Ura (0.75) [60] and 2t-Thy nucleobase and nucleotides (0.9–1) [62,63,64] contrast with the moderate singlet oxygen yields 0.36 [62,63,64] measured for them (See Table 3). The potential competition between energy transfer and charge transfer complexes has been suggested as one of the most plausible arguments for explaining such modest singlet oxygen yields [63].

#### 2.2.2. 4-Thiopyrimidines

##### 4t-Thy/Thd and 4t-Ura/Urd

• Steady State Absorption and Emission properties

The experimental absorption spectrum of 4t-Ura shows a main band in the 300–400 nm region with a maximum at ca. 328 nm (3.78 eV) in water and in ACN (see Table 6) [61,84,99]. The comparison with experiments performed on 4t-Urd in water solution reveals that the presence of the sugar moiety slightly red-shifts the maximum to ~330 nm, being again not affected by the change of the solvent (ACN) [65,72,84,100,101]. Similar absorption spectra were recorded for thiouracil methyl-derivatives (dm-4tUra) [65,102,103]. The same trends have been reported for the absorption spectra of 4t-Thd, i.e., with a maximum at 335 nm (3.70 eV) [64,67,103] in PBS, which is almost unaffected by the change of the solvent (ACN, 337 nm) [66,68].

Different computational approaches have been applied to predict the absorption spectrum of 4-thioderivatives (Table 7). Dynamically correlated methods (MCQDPT2) deliver for 4t-Ura an energy for the first ππ* state of 3.90 eV (318 nm), which is on top of the experimental value [104]. In contrast, higher energies 298–294 nm (4.16–4.22eV) are provided by monoconfigurational methods, such as TD-B3LYP and EOM-CC2 calculations [31,99,103]. Notably, other more restrictive semi-empirical methods such as INDO/S also find energies for the bright states of 4t-Ura and dm-4tUra close to experimental maximum [65].

The absorption spectra of 4t-Thd and 4t-Thy were also investigated from a computational point of view, both in GP and in solution. MS-CASPT2 calculates the spectroscopic state of 4t-Thy (S_2_) at 3.87–4.00 eV (320–310 nm) with a ππ* character [83,93]. Including explicit water molecules in the MS-CASPT2 calculation leads to a red-shift of the S_2_ energy by −0.07 eV (326 nm) [83]. QM(CASPT2)/MM calculations on 4t-Thd provide the same assignment of the bright state, also absorbing at 4.11 eV (302 nm) [95].

The spectroscopic state of 4t-Thy/4t-Thd is still S_2_ at the TD-B3LYP level of theory and involves an excitation from the HOMO to the LUMO orbitals (Figure 1). The energies of this state range from ~4.3 eV (~290 nm) in the GP to 4.15 eV (~300 nm) in ACN (PCM calculations) and decrease to 312 nm (3.97 eV) with DFT/MRCI or when the M06 functional is used [31,37,66,67,69,93,99]. 

All theses computational studies characterize the first excited state (S_1_) of 4t-Thy as a dark state which promotes an electron from the LP of the S atom to the LUMO orbital (Table 7).

• Static description of the PES

A summary of the main deactivation pathways described for 4-thioderivatives is depicted in Figure 4 (top right). Upon excitation of 4t-Thy/4t-Thd at ~330 nm, the spectroscopic state S_2_ (ππ*), as described above, is mainly populated and its deactivation mechanism has inspired several theoretical studies. Two different deactivation routes have been proposed based on MEPs performed on 4t-Thy coordinated to several explicit water molecules at the MS-CASPT2/CASSCF level of theory [83]. Both pathways converge to singlet ππ* minima, characterized by markedly different properties in terms of energy and geometry. The first S_2P_ minimum exhibits a planar conformation of the pyrimidine ring and lies lower in energy (3.49 eV), whereas the second S_2T_ is distorted along the C5=C6 double bond (recall labeling in Scheme 1) and lies much higher in energy (4.34 eV) [83]. A third singlet minimum was found in this study along the S_1_ PES presenting nπ* character (2.53 eV) [83]. Accessing the nπ* minimum requires, however, reaching the ππ*/nπ* IC funnel (4.47 eV), the efficiency of this pathway being dictated by the distribution of the trajectories between the two ππ* minima: S_2P_ is well-separated from the CoIn and should be stable against its depopulation; in contrast, the proximity of S_2T_ to the IC funnel indicates that this minimum is prone to decay to lower-lying singlet minimum [83]. Excited state optimizations of 4t-Thd at the QM(CASSCF)/MM level of theory localize, instead, two singlet excited minima, the S_2-MIN_ and S_1-MIN_ placed 3.11 eV and 2.54 eV relative to the GS equilibrium minimum, respectively [95]. The latter resembles the nπ* minimum, but the former seems not to correspond to any of the (ππ*) minima described above, i.e., it is geometrically close to the S_2_/S_1_ IC funnel in agreement with the CASPT2 S_2T_ minimum, but its low energy is in line with that of the S_2P_ minimum [95].

According to the most accurate quantum mechanical calculations, the S_2_ PES of 4t-Thy/4t-Thd presents a complicated topography governed by several local minima, which are expected to decay to lower-lying states within different time scales. 

Some experimental hints support these theoretical findings. First, although small (<1 × 10^−4^), 4t-Thd shows a measurable fluorescence quantum yield, which is consistent with the presence of a quite stable singlet minimum that is partially populated [4,67,68,83]. The nature of this emissive state has been intensively debated. Reichardt et al. [67] first assigned it to either S_1_ or S_2_ states, whereas later Wenska et al. [103] suggested that fluorescence should come from the S_2_ state, consistently with a non-Kasha behavior. Recently, Martínez-Fernández et al. pointed the S_2P_ minimum as the responsible for this fluorescence, based on the large energy required for the deactivation from this state [83]. Importantly, up-conversion experiments reported a bi-exponential decay for the fluorescence, compatible with the intricate topography of the S_2_ PES governed by different excited minima [4,83].

The fluorescence yield for 4-thiopyrimidines is very small and most of the population is expected to non-radiatively decay from the S_2_ ππ* to the dark S_1_ nπ* minimum. Once in this minimum, deactivation to the GS seems improbable since all computational studies find a large activation energy to reach the S_1_/S_0_ funnel (1.5–2.0 eV) [83,95]. As an alternative, efficient population of the triplet manifold from the nπ* minimum has been proposed due to the presence of singlet/triplet MECP near the nπ* minimum. MS-CASPT2/CASSCF MEPs locate the S_1_(nπ*)/T_1_(ππ*) crossing at the position of the S_1_ (nπ*) minimum and the QM(CASPT2/CASSCF)/MM results within less than 1 kcal·mol^−1^ from it [83,95]. Furthermore, the calculated SOCs at this region of the PES are large 60–150 cm^−1^ reinforcing the hypothesis that considers the nπ* minimum as a doorway for triplet population [69,83,93,95]. Additional singlet/triplet funnels, between the S_2_ (ππ*) and T_2_ (nπ*) states, have been also localized close in energy to the S_1_/S_2_ IC funnel [69,83,95]. The SOC at this point was calculated to be of the same order of magnitude (90–120 cm^−1^) as for the S_1_(nπ*)/T_1_(ππ*) crossing, which a priori prevents discarding deactivation from this singlet-triplet funnel, at least from a static point of view (see below) [69,83,95]. Independently of the proposed route for triplet population, all the studies conclude that the most stable T_1_ (ππ*) state (2.28–2.34 eV) will be finally populated and that its decay to the GS is not energetically favored [83,95]. Consistently with the existence of these stable triplet minima, 4t-Thd does present some phosphorescence, although the yield is small 3 × 10^−4^ [66,67,101,103]. The phosphorescence spectrum has a maximum at ~540 nm [66,69,101,102,103], matching the calculated energies for the T_1_ (ππ*) minimum both at TD-DFT (538–557 nm) [66,67,69] and CASPT2 (570 nm) levels of theory [83].

• Photodynamics

Time resolved experiments on 4t-Thd have essentially focused on predicting the ISC rates and yields in different solvents. The experimental ISC rates greatly vary depending on the solvent and experimental conditions (i.e., excitation wavelength, laser resolution, etc.). In buffer solution, experimental ISC lifetimes range from 250 fs [67,83] to 10 ps [69], whereas under the same experimental conditions they are longer in ACN, 540 fs (see Table 3) [68,83]. Interestingly, however, all the experiments uniformly determine a quantum yield for T_1_ population close to unity and a very long lifetime, from tens of ps to μs depending on the solvent, for this triplet state [66,67,68,83,101,102,103].

As far as the TAS are concerned, Reichardt et al. [67,68] discuss two peaks growing simultaneously at ~380 and 550 nm both in water and in ionic liquids, the latter also observed by Harada et al. [69]. The TAS signal peaking at ~550 nm was ascribed to the final triplet state, due to its resemblance with the triplet-triplet absorption band. Importantly, just recently an additional spectral feature in the 650–700 nm region, which was before overlooked and which decays in few ps, has been assigned to one of the two S_2_ minima, revealing important details of the relaxation mechanism [83].

From a computational point of view, the experimental τ_ISC_ = 250 fs in water is well reproduced by a simple qualitative approach, which by considering the SOC terms for the S_1_(nπ*)/T_1_(ππ*) crossing calculates a τ_ISC_ for 4t-Thy of 275 fs. [93]. However, the very different τ_ISC_ values experimentally registered suggest a more complex ISC mechanism, also in agreement with the already mentioned bi-exponential behavior of the fluorescence, (τ_1_ = 1 ps and τ_2_ = 3.6 ps) [83]. In this respect, QM/MM MD performed on 4t-Thd in solution shows that upon excitation the system actually explores regions of the PES corresponding to the planar and the twisted S_2_ minima [83]. It is actually not possible to distinguish the trajectories following the paths related to either one of these two minima, i.e., all of them seem to explore both regions of the S_2_ PES. These dynamics simulations are, however, able to discern the most likely ISC channel, the S_1_(nπ*)/T_1_(ππ*), followed by 87% of the trajectories.

This mechanism is actually supported by the results from the experimental TAS, which were interpreted with the help of MS-CASPT2 vertical spectra at the position of excited state minima. The S_2T_ minimum was found to absorb at 600 nm, whereas the S_2P_ minimum shows a maximum above 650 nm, coinciding with the spectral feature in the experimental TAS recently rediscovered at long wavelengths. Indeed, Martínez-Fernández et al. [83] showed that in order to reproduce the experimental TAS at 0.8 ps a significant population (~40%) must be still present at these minima. This assignment confirms the results of the MD simulations, which situate the photoexcited molecules in planar and twisted regions of the S_2_ PES along their relaxation to the triplet manifold (see Figure 4) [83]. Experimentally two different τ_ISC_ rates; one in the ultrafast timescale (τ_1_ = 0.54 ps) and another one in the picosecond regime (τ_2_ = 1.8 ps) were registered (see Figure 3) [83]. The long-lived signal at 550 nm was ascribed to a superposition of the two triplet minima. 

In short, the deactivation mechanism leading to triplet population in 4t-Thd is complex. Indeed, the intricacy of the PES of 4-thionucleobases is responsible for the modulation of the population rate of the T_1_ minimum and thus of the different ISC lifetimes experimentally registered.

#### 2.2.3. 2,4-Dithiopyrimidines

##### Steady State Absorption and Emission properties

The experimental absorption spectra of doubly-thionated bases (Table 8) are characterized by maxima peaking at ~360–340 nm (3.44–3.65 eV) independently of the solvent used, which supposes a red-shift compared to 4-thiopyrimidines (~320 nm) [61,64,84]. Theoretically (Table 9), the first bright state has been predicted in GP at ~330 nm with MS-CASPT2 [93], ~340 nm with DFT/MRCI [93], and 349 nm with TD-B3LYP [31], whereas in water solution the computed wavelengths are ~330 nm at MS-CASPT2 [96], 335 nm at TD-B3LYP [31] and 340 nm at the TD-M06 level of theory [37]. Equivalent calculations for 4t-Thy provided the following values 310, 312, 300 and 311 nm, all the methods being able to reproduce the red-shift of the first absorption band when both carbonyl groups are exchanged by thiocarbonyl groups [31,37,93,95]. The analysis of the molecular orbitals in the series 2t-Thy, 4t-Thy, and 2,4-dtThy performed by Bai et al. concludes that the number and position of sulfur functionalization in the pyrimidine ring leads to different electron density delocalization conditioning the orbital energies and thus the spectra [93].

##### Static description of the PES

The enhanced photosensitizing properties of 2,4-dtUra/2,4-dtThy [61,64] compared to their monosubstituted counterparts stimulated other theoretical studies, mostly focused on how triplet population occurs in these nucleobases. Following the population of the spectroscopic S_2_ (π_S2_π_4_*) state (3.62–3.72 eV) in GP, MS-CASPT2 studies (based on CASSCF geometry optimizations) predict 2,4-dtThy to undergo either adiabatic relaxation to its minimum or a barrierless IC towards low-lying S_1_ minimum (n_S2_π_2_*), (See Figure 4, bottom) [96]. The population following the first route and reaching the ππ* minimum is expected to eventually relax to the ^1^n_S2_π_2_* minimum at some stage [96]. Due to the proximity of the ^1^n_S4_π_4_*/^1^n_S2_π_2_* crossing to the ^1^n_S2_π_2_* minimum, the authors suggest that another dark minimum, presenting ^1^n_S4_π_4_* character, can also be populated [96]. Access to the GS via IC along the singlet manifold is unfortunately not explored in this work.

At the position of both ^1^n_S2_π_2_* and ^1^n_S4_π_4_* minima, MS-CASPT2//CASSCF calculates very close ISC funnels to the triplets ^3^π_S2_π_2_* and ^3^π_S4_π_4_*, the latter being strongly coupled (SOC amounting to 154 cm^−1^) to the ^1^n_S4_π_4_* state at its minimum position [96]. This value is in agreement with other SOC values obtained in GP MS-CASPT2 and MRCI studies, 152 cm^−1^ [93]. 

Xie et al. [96] paid special attention to the effect that microsolvation and aqueous solution environments have in the computed deactivation pathways of 2,4-dtThy. To this aim, they performed QM(CASSCF)/MM geometry optimizations followed by MS-CASPT2 single points in order to describe the potential energy profiles. At the FC region, water was found to destabilize the (^1^n_S2_π_2_*) dark state, decreasing the energy gap with the bright (^1^π_S2_π_4_*) spectroscopic state, thus, facilitating IC between both states. Then the authors suggest that both the ^1^n_S2_π_2_* and ^1^π_S2_π_4_* minima are populated in solution from the FC region [96]. From either singlet minima, Xie et al. [96] set out an intricate network of possible mechanisms on the way to the most stable ^3^π_S4_π_4_* minimum. However, in contrast to their GP results, in solution the population reaching the ^1^π_S2_π_4_* minimum is not assumed to later decay to the ^1^n_S2_π_2_* singlet minimum, but only to the triplet states. SOCs are not much affected by solvent [96], in agreement with Ref. [93]. Thus, water solvation was found to “entangle” the mechanism leading to the population of the ^3^π_S4_π_4_* minimum, but it is not expected to affect the final outcome of the dynamics.

##### Photodynamics

From an experimental point of view, doubly-thionated pyrimidines show very rich photophysical properties such as faster ISC rates (180 fs, 2,4-dtThy) and higher singlet oxygen quantum yield (0.49, 2,4-dtUra) compared to their monosubstituted counterparts (see Table 3) [61,64].

To the best of our knowledge, no molecular dynamics simulations are available in the literature for 2,4-dtThy/2,4-dtUra, except for a single study calculating ISC rates for 2,4-dtThy based on SOC terms [93]. The predicted ISC rate is 295 fs, which is significantly faster than the one calculated for 2t-Thy (574 fs), in agreement with the experimental findings [93]. 

Theoretical works have reported energetically accessible ISC funnels, large SOCs and stable triplet minima for 2,4-dtThy [93,96]. These features, however, are also fingerprints of the mono-substituted thiobases’ PES, what leaves open the question as to why double-thionation leads to shorter ISC lifetimes and larger ^1^O_2_ yields. A complete understanding of the photophysics of doubly-thionated pyrimidines would benefit from comprehensive molecular dynamics studies considering both singlet and triplet manifolds, and including TAS interpretation by means of theoretical calculations. 

## 3. General Remarks and Perspectives

**Figure 5:**
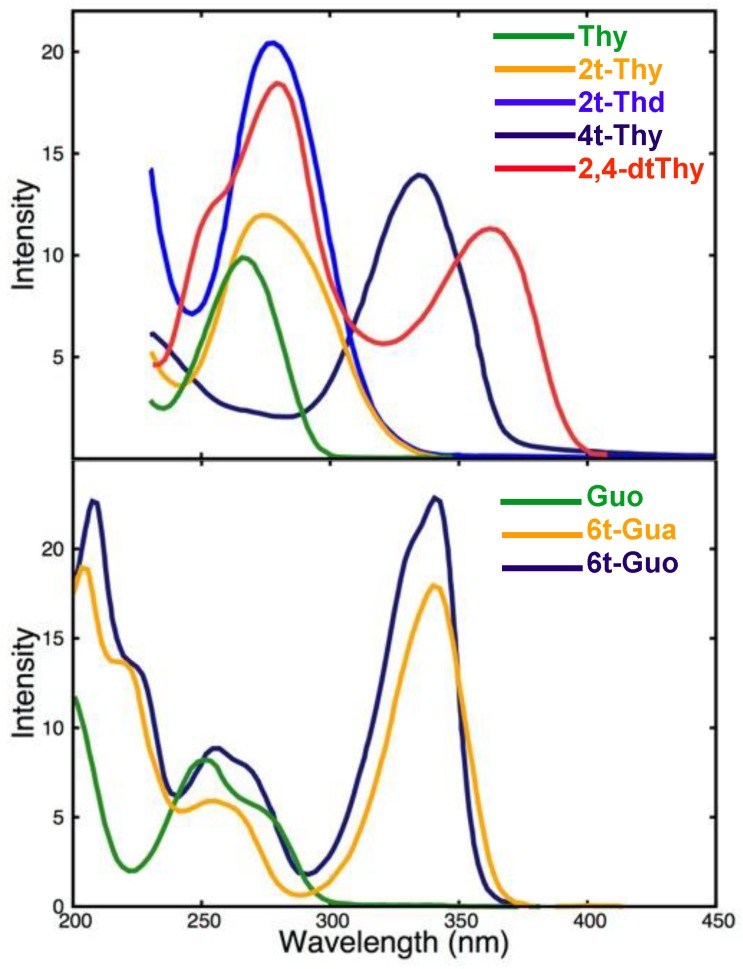
Experimental absorption spectra recorded in PBS, pH = 7.4 for Thy, Guo and their thioderivatives. The data for Thy and it derivatives (**Top**) was taken from Ref. [64]. The data for Guo and 6t-Guo (**Bottom**) was taken from Ref. [73] and the spectrum for 6t-Gua was extracted from Ref. [52].

The main effects induced by thionation in the natural DNA and RNA nucleobases’ photophysics are nowadays well established. First, exocyclic oxygen by sulfur substitution significantly red-shifts the maximum of the absorption spectrum. In contrast to natural DNA and RNA nucleobases, which are potent UVB chromophores, thiated nucleobases absorb maximally in the UVA region of the electromagnetic spectrum, (see Figure 5) being this an important side effect in pharmacological applications due to the large amount of UVA photons that reach the Earth’s surface.

Interestingly, experiments and theoretical simulations have shown that for pyrimidines the magnitude of this shift depends on the position and degree substitution, being modest in case of 2-thiopyrimidines but meaningful for 4- and 2,4-dithioderivatives [61,64]. Although calculations were found to overestimate the shifts in the absorption bands for 2-thiopyrimidines, they have provided useful hints to understand the changes in the absorption spectra from an electronic point of view. Bai et al. [93] actually succeeded to explain the observed spectral shifts on the basis of the different delocalization of the electron density provoked by the degree and pattern of substitution and the changes in the energies of the molecular orbitals involved in the absorption bands of 2t-Thy, 4t-Thy and 2,4-dtThy. It is also important to note that these shifts do not affect to the same extent all the electronic states and can therefore lead to a change of the electronic state ordering compared to canonical nucleobases. In fact, the lowest lying electronic state in all the thioderivatives presented in this review corresponds to a dark (S_1_ nπ*), something not common in canonical nucleobases [42].

**Figure 6:**
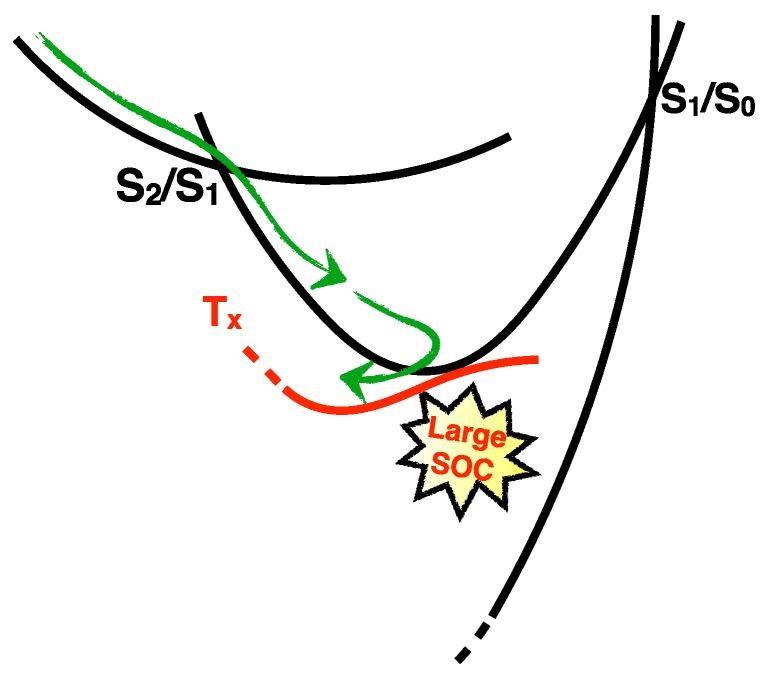
General scheme summarizing the preferred route towards the population of triplet states upon UVA excitation in thiated nucleobases.

Importantly, the exchange of one or several carbonyl groups by thiocarbonyl groups in natural nucleobases undoubtedly conditions the topography of their PES and thus of their photoinitiated dynamics. As a consequence of the important shift of the first nπ* state towards lower energies and below the spectroscopic ππ* electronic state, the first step in the relaxation mechanisms of all canonical nucleobases’ thiated analogs is an IC process towards the first excited PES. This deactivation channel has been shown to be preferred to direct ISC from the S_2_. The main differences between thiated and natural nucleobases lie on the subsequent relaxation from S_1_. Even though recent studies in solution have located small energy barriers associated to GS deactivation from the S_1_, it has been generally assumed that this is the most favorable mechanism in canonical nucleobases [42,106]. By contrast, this deactivation route is strongly prevented in sulfur-substituted nucleobases since the S_1_/S_0_ funnels are energetically inaccessible due to the significant stabilization of the S_1_ minima (Figure 6). Furthermore, another secondary effect of sulfur substitution is that other S_1_/S_0_ CoIn different to the preferred ones for canonical nucleobases may come into play [34]. Besides, oxygen by sulfur substitution also provokes a decrease in the singlet-triplet energy gaps and the enhancement of the SOC (thiobases 100–200 cm^−1^; canonical bases < 50 cm^−1^) due to the heavy atom effect of S. Both effects are translated into energetically accessible and very efficient singlet-triplet crossings at the vicinity of the S_1_ minima of thioderivatives, which correspond to secondary routes in canonical nucleobases (Figure 6). Indeed, ultrafast ISCs and close to unity triplet yields were experimentally observed and inferred from MD simulations [4,34,58,83,98]. Although Pollum et al. [61,64] have registered enhanced photosensitizing properties in doubly thionated nucleobases, the reasons for their faster ISC and closer to unity triplet yields are still unclear since singly- and doubly-substituted thiobases share the main features of the PES. The long life of their most stable triplet state, characterized in the case of 2t-Thy, 2t-Ura, 6t-Gua, 2t-Cyt by double well potential energy profile, is also common to all the thiobases [55,59,89]. 

Thiobases are known to be important precursors of ^1^O_2_ in solution via Type II photosensitizing mechanisms, with yields comprised between 0.14 and 0.58 (see Table 3) [4,73]. The significant differences between the triplet and singlet oxygen quantum yields suggest that other mechanisms, such as charge transfer reactions with O_2_, might compete in solution [62,63,65,73,83]. In cellular or biological environments, the situation is much more complex since Type I and Type II mechanisms can involve a broad spectrum of biomolecules leading to UVA induced lesions including canonical nucleobases’, 6t-Gua’s and protein’s oxidation products, DNA and DNA–protein crosslinks and DNA strand breaks [16,79,82,107].

Although the basis for the first stages of the photosensitizing reactions of thiated nucleobases that lead to triplet excited states’ population have been clearly established by experimental and computational works, a great piece of work still remains to be done for the characterization of the mechanisms at molecular level leading to photoproducts’ formation, which are ultimately responsible for the mechanism of action of these photosensitizers in medicinal chemistry.

## Figures and Tables

**Table 1:** Steady-state absorption and emission properties of 6-thioguanine nucleobase and nucleosides at room temperature unless otherwise indicated. F: Fluorescence; P: Phosphorescence; N.D.: Not detected.

	λ_max_ (nm)	ε_max_ (M^−1^ cm^−1^ × 10^4^)	λ_emission_ (nm)		Solvent
			F	P	
6t-Gua	337	1.80	–	–	NaOH, pH = 10 [56]
	340	2.10	–	–	Tris buffer, pH = 7.4 [56]
	340, 255, 220, 205	–	400	–	H_2_O, pH = 2.3 [51]
	340, 255, 220, 205	–	400, 500 ^1^	–	H_2_O, pH = 7.8 [51]
	320, 265, 245, 210	–	400	–	H_2_O, pH = 12.8 [51]
	341, 254, 220 ^1^, 204	1.80, 0.60, 1.40, 1.90	–	–	PBS, pH = 7.4 [52]
	347 ^2^	2.08	–	478	pH = 1.0, 77 K [50]
	343 ^2^	2.22	–	459	pH = 5.0, 77 K [50]
	323 ^2^	1.85	–	442	pH = 10.0, 77 K [50]
	321 ^2^	2.12	–	442	1 N NaOH, 77 K [50]
	–	–	468, 437 ^1^, 494 ^1^	430–550	Tris buffer, pH = 7.4, 77 K [52]
	–	–	–	478	NaOH, pH = 12.1 [57]
6t-Guo	320	1.80	–	–	NaOH, pH = 10 [56]
	342	2.00	–	–	Tris buffer, pH = 7.4 [56]
	342	2.30	N.D.	N.D.	PBS, pH = 7 [54]
	346	2.20	N.D.	N.D.	ACN [54]
	341, 258, 227 ^1^, 208	2.30, 0.80, 1.20, 2.10	–	–	PBS, pH = 7.4 [52]
	348 ^2^	–	–	481	pH = 1.0 ,77 K [50]
	344 ^2^	–	–	459	pH = 5.0, 77 K [50]
	316 ^2^	–	–	431	pH = 12.0, 77 K [50]

^1^ Band shoulder. ^2^ Absorption maxima at room temperature.

**Table 2:** Computed absorption and emission properties of 6-thioguanine nucleobase and nucleosides. Only information relative to the lower lying spectroscopic state and preceding electronic states is included. In parenthesis the oscillator strengths are shown.

	λ_max_ (nm)	Solvent	Excitation	λ_emission_ (nm)		Method
				F	P	
6t-Gua [55]	369 (0.000)	–	S_1_^1^nπ*	395	–	MS-CASPT2// CASSCF(14,12)/ANO-L
	306 (0.535)	–	S_2_^1^ππ*	–	–	
	400	–	T_1_^3^ππ*	–	404	
	375	–	T_2_^3^nπ*	–	–	
6t-Gua [53]	397 (0.000)	–	S_1_	–	–	TD-B3LYP/6-311++G(d,p)
	311 (0.203)	–	S_2_	–	–	
6t-Gua [52]	381 (0.000)	–	S_1_^1^nπ*	–	–	TD-PBE0/6-311++G(d,p)/ IEFPCM
	346 (0.000)	H_2_O	S_1_^1^nπ*	–	–	
	299 (0.260)	–	S_2_^1^ππ*	–	–	
	310 (0.400)	H_2_O	S_2_^1^ππ*	–	–	
6t-Gua [50]	337 (0.470)	–	S_2_^1^ππ*	–	–	CNDO/S
6t-Gua [51]	400 (0.000)	–	S_1_^1^nπ*	–	–	CNDO/s-CI
	340 (0.290)	–	S_2_^1^ππ*	–	–	
6t-Guo [54]	384 (0.000)	–	S_1_^1^nπ*	–	–	TD-PBE0/6-311++G(d,p)/ IEFPCM
	341 (0.000)	H_2_O	S_1_^1^nπ*	–	–	
	342 (0.000)	ACN	S_1_^1^nπ*	–	–	
	305 (0.280)	–	S_2_^1^ππ*	–	–	
	313 (0.450)	H_2_O	S_2_^1^ππ*	–	–	
	313 (0.450)	ACN	S_2_^1^ππ*	–	–	
6t-Guo [38]	359 (0.000)	–	S_1_^1^nπ*	376	–	TD-B3LYP/6-31+G*
	319 (0.434)	–	S_2_^1^ππ*	–	–	
	457	–	T_1_^3^ππ*	–	496	
	357 (0.000)	–	S_1_^1^nπ*	–	–	TD-M06/6-31+G*
	314 (0.455)	–	S_2_^1^ππ*	–	–	

**Table 3:** Photophysical parameters (lifetimes in ps unless otherwise indicated and triplet and singlet oxygen quantum yields) for thiated nucleobase analogs.

	τ _ISC_ (ps)	Φ _ISC_	Lifetimes	Φ_Δ_
2t-Ura	0.35 ± 0.06 ^8^ [60]	0.75 ± 0.20 ^8^ [60]	τ_1_ < 0.20, τ_2_ = 0.35 ± 0.06 ^8^, 0.34 ± 0.09 ^1^ [60]	–
	0.34 ± 0.09 ^1^ [60]	–		–
	0.36 ± 0.03 ^8^ [61]	–	–	–
2t-Thy	0.62 ± 0.07 ^8^ [60]	1.00 ± 0.05 ^3^ [62]	τ_1_ < 0.20, τ_2_ = 0.62 ± 0.07 ^8^, 0.32 ± 0.09 ^1^ [60], τ_3_ = 2.7 ± 0.5 (μs) [62]	0.36 ± 0.02 ^3^ [62]
	0.32 ± 0.09 ^1^ [60]	0.9 ± 0.1 ^2^ [63]	τ_1_ < 0.10, τ_2_ = 0.78, τ_3_ = 203 [39]	–
2t-Thd	0.41 ± 0.06 ^8^ [64]	0.9 ± 0.1 ^2^ [63]	–	–
2t-Cyt	0.21 ± 0.05 ^8^ [34]	>0.9 ^8^ [34]	τ_1_ = 0.21 ± 0.05, τ_2_ = 0.48 ± 0.06 [34]	–
dm-4tUra	–	1.0 ± 0.1 ^9^ [65]	–	–
4t-Ura	0.24 ± 0.02 ^8^ [61]	0.9 ± 0.15 ^8^ [61]	–	0.49 ± 0.02 ^3^ [61]
4t-Thd	0.24 ± 0.02 ^8^ [64]	1.0 ± 0.1 ^2^ [66]	–	0.50 ± 0.10 ^3^ [66]
	0.24 ± 0.02 ^8^ [67]	0.85 ± 0.15 ^8^ [67]	τ_1_ = 0.24 ± 0.02, τ_2_ = 84 ± 2 [67]	0.42 ± 0.02 ^3^ [64]
	0.54 ± 0.01 ^1^ [68]	–	τ_1_ = 0.54 ± 0.01, τ_2_ = 45 ± 5 [68], τ_1_ = 0.54, τ_2_ = 1.8 [57]	–
	~10 ^1^ [69]			
4t-Urd	–	–	–	0.7 [70]
	–	0.9 ± 0.1 ^9^ [65]	–	0.18 ± 0.04 ^10^ [4,71]
	–	0.02 ^2^ [72]	–	0.50 ±0.02 ^3^ [4,71]
2,4-dtUra	0.22 ± 0.04 ^8^ [61]	0.9 ± 0.15 ^8^ [61]	–	0.49 ± 0.02 ^3^ [61]
2,4-dtThy	0.18 ± 0.02 ^8^ [64]	>0.9 ^8^ [64]	–	0.46 ± 0.02 ^3^ [64]
6t-Gua	–	–	–	0.58 ± 0.08 ^6^ [56]
	–	–	–	0.56 ± 0.18 ^5^ [56]
	0.56 ± 0.06 ^8^ [52]	≥0.6± 0.2 ^8^ [52]	τ_1_ = 0.56 ± 0.06, τ_2_ = 26 ± 3, τ_3_ = 830 ± 70 (ns), τ_3_ = 1420 ± 180 (ns) (N_2_) [52]	0.21 ± 0.02 ^4^ [52]
	–	–		0.23 ± 0.02 ^5^ [52]
6t-Guo	0.31 ± 0.05 ^7^ [54]	0.8 ± 0.2 ^7^ [54]	τ_1_ = 0.31 ± 0.05, τ_2_ = 80 ± 15, τ_3_ = 460 ± 15 (ns), τ_3_ = 720 ± 10 (ns) (N_2_) [54]	0.49 ± 0.09 ^6^ [56]
	–	–	–	0.24 ± 0.02 ^5^ [73]
	–	–	–	0.14 ± 0.02 ^4^ [73]
	–	–	–	0.29 ± 0.02 ^3^ [73]
	0.36 ± 0.04 ^1^ [54]	–	τ_1_ = 0.36 ± 0.04, τ_2_ = 32 ± 5 [54]	0.55 ± 0.08 ^5^ [56]

**^1^** ACN; ^2^ ACN, Ar; ^3^ ACN, O_2_; ^4^ D_2_O, Tris buffer, pH = 7.4; ^5^ D_2_O, Tris buffer, pH = 7.4, O_2_; ^6^ NaOH/D_2_O, pH = 10; ^7^ PBS, pH = 7.0; ^8^ PBS, pH = 7.4; ^9^ H_2_O, Ar, ^10^ H_2_O, O_2_.

**Table 4:** Steady-state absorption and emission properties of 2-thiopyrimidine derivatives at room temperature unless otherwise indicated. F: Fluorescence; P: Phosphorescence; N.D.: Not detected.

	λ_max_ (nm)	ε_max_ (M^−1^ cm^−1^ × 10^4^)	λ_emission_ (nm)		Φ (×10^−4^)		Solvent
			F	P	F	P	
2t-Ura	274	1.45	–	–	–	–	H_2_O, pH = 7 [84]
	268	1.19	–	–	–	–	ACN [84]
	274, 291 ^1^	_	–	–	–	–	MeOH [84]
	271, 290 ^1^	–	N.D.	405, 427, 455	–	7.0	EtOH, 77 K [86]
	265	–	–	–	–	–	PBS, pH = 7.4 [61]
2t-Urd	275	1.67	–	–	–	–	H_2_O, pH = 7 [84]
	268	1.04	–	–	–	–	ACN [84]
2t-Thy	275, 290 ^1^	1.29, 1.33	N.D.	N.D.	–	–	ACN [62]
	–	–	–	454	–	–	THF, 77 K [62]
	290	1.24	N.D.	480	–	5.0, 9.8 ^2^	ACN, Ar [63]
	–	–	–	448	–	–	DCM:MeOH, 77 K [63]
	275, 290 ^1^	–	N.D.	425, 451, 483	–	7.0	EtOH, 77 K [86]
	275	–	–	–	–	–	PBS, pH = 7.4 [64]
2t-Thd	285	1.41	N.D.	N.D.	<1 ^3^	–	ACN, Ar [63]
	–	–	–	448	–	–	DCM:MeOH, 77 K [63]
	277	–	–	–	–	–	PBS, pH = 7.4 [64]
2t-Cyt	270, 242, 220 ^1^	1.82	–	–	–	–	H_2_O, pH = 7 [84]
	269, 241, 219 ^1^	–	–	–	–	–	PBS, pH = 7.4 [34]
	283, 233	1.67, 1.19	–	–	–	–	ACN [85]
	286	1.69	–	–	–	–	EtOAc [85]
	285	1.80	–	–	–	–	DMSO [85]
	280, 242, 210 ^1^	1.79, 1.42, 0.65	–	–	–	–	EtOH [85]
	278, 242, 212 ^1^	1.78, 1.48, 0.71	–	–	–	–	MeOH [85]
	269, 242, 218 ^1^	1.80, 1.80, 0.90	–	–	–	–	PBS, pH = 7.4 [85]
5m-2tCyt	266	2.10	–	–	–	–	H_2_O, pH = 7 [84]
2t-Cyd	274, 270 ^1^	2.30, 1.40	–	–	–	–	H_2_O, pH = 7 [84]

**^1^** Band shoulder. ^2^ Calculated to infinite dilution. ^3^ Estimated due to the sensitivity limit of the instrument.

**Table 5:** Computed absorption and emission properties of 2-thiopyrimidine derivatives. Only information concerning the lowest lying spectroscopic state and preceding states is included. In parenthesis the oscillator strengths are shown. F: Fluorescence; P: Phosphorescence.

	λ_max_ (nm)	Solvent	Excitation	λ_emission_ (nm)		Method
				F	P	
2t-Ura ^1^ [89]	329 (0.000)	–	S_1_^1^nπ*	358	–	MS(6/4)-CASPT2/ANO-RCC-VQZQ// SA(6/4)-CASSCF(16,12)
	292 (0.350)	–	S_2_^1^ππ*	–	–	
	353	–	T_1_^3^ππ*	–	386	
	322 (0.000)	–	S_1_^1^nπ*	–	–	MRCIS(6,5)/cc-pVDZ// SA(4/2)-CASSCF(12,9)
	215 (0.110)	–	S_2_^1^ππ*	–	–	
2t-Ura ^1^ [90]	323	–	S_1_^1^nπ*	325	–	CASPT2/cc-pVDZ// SA(4/3)-CASSCF(16,11)
	278	–	S_2_^1^ππ*	–	–	
	–	–	T_1_^3^ππ*	–	358	
	347	–	S_1_^1^nπ*	–	–	TD-B3LYP/aug-cc-pVDZ
	304	–	S_2_^1^ππ*	–	–	
2t-Ura [91]	340 (0.000)	–	S_1_^1^nπ*	369	–	SS-CASPT2/ANO-L-vDZP// SA(6/6)-CASSCF(14,10)
	303 (0.236)	–	S_2_^1^ππ*	349	–	
	395	–	T_1_^3^ππ*	–	435	
2t-Ura [31]	346 (0.000)	–	S_1_^1^nπ*	–	–	TD-B3LYP/6-311++G(d,p)
	304 (0.051)	–	S_2_^1^ππ*	–	–	
	317 (0.000)	H_2_O	S_1_^1^nπ*	–	–	
	286 (0.145)	H_2_O	S_2_^1^ππ*	–	–	
	318 (0.000)	ACN	S_1_^1^nπ*	–	–	
	287 (0.144)	ACN	S_2_^1^ππ*	–	–	
2t-Ura [92]	320 (0.000)	ACN	S_1_^1^nπ*	–	–	TD-B3LYP/6-31+G(d)/PCM
	283 (0.108)	ACN	S_2_^1^ππ*	–	–	
2t-Ura [86]	332 (0.000)	EtOH	S_1_^1^nπ*	–	–	TD-B3LYP/aug-cc-pVDZ/PCM
	298 (0.110)	EtOH	S_2_^1^ππ*	–	–	
2t-Thy [37]	325	H_2_O	S_1_^1^nπ*	–	–	TD-M06/6-31+G*/PCM
	288	H_2_O	S_2_^1^ππ*	–	–	
2t-Thy ^1,2^ [93]	286 (0.578)	–	^1^ππ*	–	–	MS-CASPT2(14,10)/ANO-RCC-VTZP
	295 (0.243)	–	^1^ππ*	–	–	DFT/MRCI
2t-Thy [62]	315 (0.000)	ACN	S_1_^1^nπ*	–	–	TD-B3LYP/6-31+G(d,p)/PCM
	295 (0.145)	ACN	S_2_^1^ππ*	–	–	
2t-Thy [86]	323 (0.000)	EtOH	S_1_^1^nπ*	–	–	TD-B3LYP/aug-cc-pVDZ/PCM
	301 (0.125)	EtOH	S_2_^1^ππ*	–	–	
2t-Thy [53]	339 (0.000)	–	S_1_	–	–	TD-B3LYP/6-311++G(d,p)
	308 (0.054)	–	S_2_	–	–	
2t-Cyt ^1^ [34]	340 (0.001)	–	S_1_^1^nπ*	420	–	MS-CASPT2/CASSCF(14,10)/ ANO-RCC-VQZP//RI-MP2/cc-pVQZ
	332 (0.097)	–	S_2_^1^ππ*	410	–	
	368	–	T_1_^3^ππ*	–	435	
2t-Cyt [85]	358 (0.000)	–	S_1_^1^nπ*	–	–	MS-CASPT2(14,10)/cc-pVDZ/PCM//BP86/aug-cc-pVDZ/COSMO
	315 (0.000)	EtOAc	S_1_^1^nπ*	–	–	
	309 (0.000)	ACN	S_1_^1^nπ*	–	–	
	309 (0.000)	DMSO	S_1_^1^nπ*	–	–	
	286 (0.000)	EtOH	S_1_^1^nπ*	–	–	
	284 (0.000)	MeOH	S_1_^1^nπ*	–	–	
	276 (0.020)	H_2_O	S_1_^1^nπ*	–	–	
	357 (0.020)	–	S_2_^1^ππ*	–	–	
	315 (0.010)	EtOAc	S_2_^1^ππ*	–	–	
	311 (0.020)	ACN	S_2_^1^ππ*	–	–	
	307 (0.020)	DMSO	S_2_^1^ππ*	–	–	
	294 (0.030)	EtOH	S_2_^1^ππ*	–	–	
	292 (0.030)	MeOH	S_2_^1^ππ*	–	–	
	293 (0.010)	H_2_O	S_2_^1^ππ*	–	–	
2t-Cyt [53]	403 (0.000)	–	S_1_	–	–	TD-B3LYP/6-311++G(d,p)
	371 (0.010)	–	S_2_	–	–	

^1^ This work reports additional values obtained at other levels of theory. ^2^ This work reports values computed considering other solvents.

**Table 6:** Steady-state absorption and emission properties of 4-thiopyrimidine derivatives at room temperature unless otherwise indicated. F: Fluorescence; P: Phosphorescence; N.D.: Not detected, PFDMCH: perfluoro-1,3-dimethylcyclohexane.

	λ_max_ (nm)	ε_max_ (M^−1^ cm^−1^ × 10^4^)	λ_emission_ (nm)		Φ (×10^−4^)		Solvent
			F	P	F	P	
4t-Ura	327	1.28	–	–	–	–	ACN [84]
	328	1.67	–	–	–	–	H_2_O, pH = 7 [84]
	327	1.94	–	–	–	–	ACN [99]
	330	–	–	550	–	3.0 ^1^	H_2_O [17]
dm-4tUra	328	1.98	415	528	0.6 ^1^	14.0 ^1^	CCl_4,_ He [102]
	317	–	416	535	1.5 ^1^	25.0 ^1^	PFDMCH, He [102]
	329	1.88	420	535	0.4	–	H_2_O [102]
4t-Urd	331	2.30	–	–	–	–	H_2_O, pH = 7 [84]
	331	1.19	–	–	–	–	ACN [84]
Ac-4tUrd	328	2.06	420	550	1.0	2.0	ACN [100]
	330	2.06	420	540	–	123 ^1^	CCl_4,_ He [101]
	–	–	–	–	–	25 ^1^	ACN, Ar [105]
4t-Thd	337	–	410	550	–	–	ACN [68]
	335	–	400	542	–	–	PBS, pH = 7.4 [67]
	337	1.94	N.D.	N.D.	–	–	ACN [66]
	–	–	–	494, 512, 560 ^2^	–	–	EtOH, 77 K [66]

**^1^** Calculated to infinite dilution. ^2^ Band shoulder.

**Table 7:** Computed absorption and emission properties of 4-thiopyrimidine derivatives. In parenthesis the oscillator strengths are shown. F: Fluorescence; P: Phosphorescence.

	λ_max_ (nm)	Solvent	Excitation	λ_emission_ (nm)		Method
				F	P	
4t-Ura [31]	446 (0.000)	–	S_1_^1^nπ*	–	–	TD-B3LYP/6-311++G(d,p)
	295 (0.298)	–	S_2_^1^ππ*	–	–	
	387 (0.000)	H_2_O	S_1_^1^nπ*	–	–	
	298 (0.437)	H_2_O	S_2_^1^ππ*	–	–	
	389 (0.000)	ACN	S_1_^1^nπ*	–	–	
	299(0.440)	ACN	S_2_^1^ππ*	–	–	
4t-Ura [104]	401	–	S_1_^1^nπ*	–	–	CASSCF
	259	–	S_2_^1^ππ*	–	–	
	440	–	S_1_^1^nπ*	–	–	MCQDPT2
	318	–	S_2_^1^ππ*	–	–	
4t-Ura [99]	444 (0.000)	–	S_1_^1^nπ*	–	–	TD-B3LYP/6-311++G(d,p)
	294 (0.299)	–	S_2_^1^ππ*	–	–	
dm-4tUra [103]	408	–	S_1_^1^nπ*	–	–	EOM-CC2/aug-cc-pVDZ
	296	–	S_2_^1^ππ*	–	–	
4t-Thy ^1^ [93]	310 (0.662)	–	^1^ππ*	–	–	MS-CASPT2 (14,10)/ANO-RCC/VTZP
	312 (0.427)	–	^1^ππ*	–	–	DFT/MRCI
4t-Thy [53]	438 (0.000)	–	S_1_	–	–	TD-B3LYP/6-311++G(d,p)
	298 (0.282)	–	S_2_	–	–	
4t-Thy [37]	403	H_2_O	S_1_^1^nπ*	–	–	TD-M06/6-31+G*/PCM
	312	H_2_O	S_2_^1^ππ*	–	–	
4t-Thy [83]	419 (0.000)	H_2_O (PCM + 7 H_2_O )	S_1_^1^nπ*	–	–	MS-CASPT2 (12,9)/ANO-L
	326 (0.546)	H_2_O (PCM + 7 H_2_O )	S_2_^1^ππ*	430	–	
	448	–	T_1_^3^ππ*	–	570	
4t-Thd ^1^ [67]	385 (0.000)	H_2_O (PCM + 2 H_2_O )	S_1_^1^nπ*	–	–	TD-PBE0/IEFPCM/6-311++G(d,p)
	310 (0.509)	H_2_O (PCM + 2 H_2_O )	S_2_^1^ππ*	–	–	
	478	H_2_O (PCM + 2 H_2_O )	T_1_^3^ππ*	–	557 ^2^	
4t-Thd [66]	292 (0.379)	–	S_2_^1^ππ*	–	–	TD-B3LYP/6-31G(d,p)
4t-Thd [69]	~435	–	S_1_^1^nπ*	–	–	TD-B3LYP/6-31G(d,p)
	~294	–	S_2_^1^ππ*	–	–	
4t-Thd [95]	413	H_2_O (MM)	S_1_^1^nπ*	–	–	QM(CASPT2//CASSCF(10,8))/6-31G*/MM
	302	H_2_O (MM)	S_2_^1^ππ*	–	–	

**^1^** This work reports additional values obtained at other levels of theory and computed considering other solvents. ^2^ Calculated at the same level of theory without the explicit water molecules for 4t-Thd.

**Table 8:** Steady-state absorption and emission properties of 2,4-dithiopyrimidine derivatives at room temperature. F: Fluorescence; P: Phosphorescence.

	λ_max_ (nm)	ε_max_ (M^−1^ cm^−1^ × 10^4^)	λ_emission_ (nm)		Solvent
			F	P	
2,4-dtUra	351	0.46	–	–	H_2_O, pH = 7 [84]
	351	0.10	–	–	ACN [84]
2,4-dtUrd	345	1.14	–	–	H_2_O, pH = 7 [84]
	351	1.00	–	–	ACN [84]
2,4-dtThy	363	0.97	–	–	PBS [64]

**Table 9:** Computed absorption and emission properties of 2,4-dithiopyrimidine derivatives. In parenthesis the oscillator strengths are shown. F: Fluorescence; P: Phosphorescence.

	λ_max_ (nm)	Solvent	Excitation	λ_emission_ (nm)		Method
				F	P	
2,4-dtUra [31]	449 (0.000)	–	S_1_^1^nπ*	–	–	TD-B3LYP/6-311++G(d,p)
	349 (0.014)	–	S_2_^1^ππ*	–	–	
	396 (0.000)	H_2_O	S_1_^1^nπ*	–	–	
	335 (0.063)	H_2_O	S_2_^1^ππ*	–	–	
	399 (0.000)	ACN	S_1_^1^nπ*	–	–	
	336 (0.063)	ACN	S_2_^1^ππ*	–	–	
2,4-dtThy ^1^ [93]	333 (0.216)	–	^1^ππ*	–	–	MS-CASPT2(14,10)/ANO-RCC-VTZP
	343 (0.078)	–	^1^ππ*	–	–	DFT/MRCI
2,4-dtThy ^1^ [96]	400	H_2_O	S_1_^1^nπ*	446	–	QM(MS-CASPT2)/MM
	334	H_2_O	S_2_^1^ππ*	–	–	
	445	H_2_O	T_1_^1^ππ*	–	474	
2,4-dtThy [53]	443 (0.000)	–	S_1_	–	–	TD-B3LYP/6-311++G(d,p)
	361 (0.014)	–	S_2_	–	–	
2,4-dtThy [37]	409	H_2_O	S_1_	–	–	TD-M06/6-31+G*/PCM
	340	H_2_O	S_2_	–	–	

**^1^** This work reports additional values obtained at other levels of theory and computed considering other solvents.
